# Evolution of Pre-Existing versus Acquired Resistance to Platinum Drugs and PARP Inhibitors in BRCA-Associated Cancers

**DOI:** 10.1371/journal.pone.0105724

**Published:** 2014-08-26

**Authors:** Kimiyo N. Yamamoto, Kouji Hirota, Shunichi Takeda, Hiroshi Haeno

**Affiliations:** 1 Department of Information and Computer Sciences, Nara Women's University, Nara, Japan; 2 Japan Society for the Promotion of Science, Tokyo, Japan; 3 Department of Chemistry, Tokyo Metropolitan University, Tokyo, Japan; 4 Department of Radiation Genetics, Graduate School of Medicine, Kyoto University, Kyoto, Japan; 5 Department of Biology, Kyushu University, Fukuoka, Japan; University of Hawaii Cancer Center, United States of America

## Abstract

Platinum drugs and PARP inhibitors (“PARPis”) are considered to be effective in BRCA-associated cancers with impaired DNA repair. These agents cause stalled and collapsed replication forks and create double-strand breaks effectively in the absence of repair mechanisms, resulting in arrest of the cell cycle and induction of cell death. However, recent studies have shown failure of these chemotherapeutic agents due to emerging drug resistance. In this study, we developed a stochastic model of BRCA-associated cancer progression in which there are four cancer populations: those with (i) functional BRCA, (ii) dysfunctional BRCA, (iii) functional BRCA and a growth advantage, and (iv) dysfunctional BRCA and a growth advantage. These four cancer populations expand from one cancer cell with normal repair function until the total cell number reaches a detectable amount. We derived formulas for the probability and expected numbers of each population at the time of detection. Furthermore, we extended the model to consider the tumor dynamics during treatment. Results from the model were validated and showed good agreement with clinical and experimental evidence in BRCA-associated cancers. Based on the model, we investigated conditions in which drug resistance during the treatment course originated from either a pre-existing drug-resistant population or a *de novo* population, due to secondary mutations. Finally, we found that platinum drugs and PARPis were effective if (i) BRCA inactivation is present, (ii) the cancer was diagnosed early, and (iii) tumor growth is rapid. Our results indicate that different types of cancers have a preferential way of acquiring resistance to platinum drugs and PARPis according to their growth and mutational characteristics.

## Introduction

The inactivation of BRCA1 or BRCA2 (BRCA1/2) is considered to be an important step in the tumorigenesis of breast and ovarian cancers [1]. BRCA1/2 mutations are also found in a small proportion of prostate, pancreatic, and uterine serous cancers [2]–[4]. Loss of functional BRCA is strongly associated with the incidence of BRCA-associated cancers, such as basal-like breast cancer [5], [6]. Moreover, mutations in BRCA1/2 genes due to several mechanisms, such as germline mutations, somatic mutations, and epigenetic silencing, are present in 33% of ovarian carcinoma samples [7]. However, it has also become evident that biallelic loss of wild-type BRCA is not required for tumorigenesis in some types of BRCA-associated breast cancers [8]–[10]. Consistently, loss of wild-type BRCA1 is not the initiating step in tumorigenesis in BRCA-associated breast tumors [11]. Additionally, a high level of heterogeneity in loss of heterozygosity (LOH) was observed in breast cancer with BRCA1/2 heterozygotes [12]. These lines of evidence indicate that BRCA-associated cancers undergo two different types of evolutionary trajectories: tumorigenesis with loss of both BRCA alleles and tumorigenesis with BRCA heterozygosity. Other genes, such as TP53 and PIK3CA, are also mutated in BRCA-associated cancers [5]. These mutations confer growth advantages on cancer cells and drive tumorigenesis [13], [14].

The BRCA1/2 proteins have essential functions in preserving chromosomal integrity during cell division. DNA replication forks frequently stall even during normal cell proliferation and may generate DNA double-strand breaks (DSBs). These DSBs are repaired by BRCA1/2 via homologous recombination (HR) in an error-free fashion [15]. Without functional BRCA1/2, error-prone repair pathways are selectively stimulated, provoking genetic instability [16], [17]. Such genetic instability does not confer growth advantages to cells but accelerates the process of genetic variation that drives carcinogenesis by inducing additional mutational events [18]. Moreover, statistical analyses have shown that there is a correlation between high mutation frequency and DNA repair pathway genes, such as BRCA1/2 [19].

Currently, platinum-based therapy is a major option for BRCA1/2-mutated tumors, such as ovarian cancer [20]. Platinum drugs, such as cisplatin and carboplatin, induce interstrand cross-links (ICLs), inhibiting cellular replication and transcription. BRCA1/2-deficient cells are particularly sensitive to ICL-inducing agents because ICLs are repaired through a Fanconi anemia/BRCA pathway [21]. Several studies indicate that ovarian cancer patients with BRCA-germline mutations show favorable responses to platinum drugs [7], [22], [23]. Moreover, poly ADP-ribose polymerase (PARP) inhibitors (PARPis) have gained attention as effective drugs for BRCA-mutated cancers [24]. PARPis leave single-strand breaks (SSBs) unrepaired and induce DSBs. Cancer cells deficient in BRCA1/2 are unable to maintain genomic integrity in the presence of a large number of DSBs, resulting in cell death via a synthetic lethal effect. Cells carrying BRCA mutations are up to 1,000-fold more sensitive to PARPis than wild-type cells [25]. Finally, multiple PARPis are currently in clinical development for cancers deficient in the Fanconi anemia/BRCA pathway [24].

However, chemotherapy using platinum drugs or PARPis often fails because of the emergence of resistance; indeed, most patients will ultimately have refractory disease [20], [24]. Several mechanisms of resistance to platinum drugs have been identified: (i) mutations in cell-membrane transport proteins decrease drug uptake, resulting in reduced intracellular platinum concentrations, (ii) mutations in apoptotic signaling pathways prevent a cell from inducing cell death, and (iii) back mutations to wild-type BRCA1/2 result in the restored ability to repair DNA damage generated by platinum drugs [26], [27]. Clinical studies have also identified a major mechanism of resistance to PARPis, in which secondary mutations restore BRCA function [28]–[30].

Resistant mutations can arise either prior to or during chemotherapy. On the one hand, resistant cells may pre-exist in a tumor before treatment and expand under selective pressure after treatment initiation. Indeed, it has been shown that platinum-sensitive and -resistant cells shared a common ancestor during the early stages of tumor development [31]. On the other hand, resistant cells may emerge as a result of novel mutations during treatment and expand under the selective pressure of treatment. The acquisition of secondary mutations has been observed with platinum drug and PARPi treatment [27], [28]. Because the emergence of such resistance leads to treatment failure, it is important to investigate conditions in which resistant cells exist before treatment and appear after treatment.

Mathematical investigations have provided insights into how tumor cells drive progression and acquire drug resistance by accumulating mutations. Recently, the emergence of drug-resistant cancer cells from one specific mutation during clonal expansion prior to treatment was considered [32]. Moreover, the evolutionary dynamics of BRCA1-mutated breast cancer initiation were also considered, with the assumption that the number of cells is constant [33]. Breast cancer development caused by inactivation of two tumor suppressor genes has also been investigated [34]. In the case of ovarian cancer progression, a branching process model, accounting for primary, peritoneal, and metastatic cancer populations, was evaluated [35]. Furthermore, the evolution of resistance in cancer cells during continuous and pulsed administration strategies was suggested [36]. The risk of harboring multiple types of resistance at the start of chemotherapy due to various point mutations was studied in chronic myeloid leukemia [37]. In addition, the expected number of mutations conferring drug resistance in colorectal cancer was estimated using a branching process model [38]. Our study is based on a foundation of many previous theoretical studies regarding the accumulation of mutations in cancer cells [39]–[43].

In this study, we investigated tumor progression mathematically and the evolution of resistance to platinum drugs and PARPis in BRCA1/2-mutated cancers before and during treatment. We focused on the specific effects caused by loss of BRCA1/2 function, which confers (epi)genetic instability in cancer cells. Cancer cells with dysfunctional BRCA1/2 acquire increased mutation rates and become sensitive to platinum drugs and PARPis due to a deficiency in error-free repair mechanisms.

First, we developed a mathematical model of BRCA-associated cancer progression, in which two types of mutations were included: (i) those conferring functional BRCA1/2 inactivation and (ii) those accelerating cell growth by inactivation of cell cycle regulation. Second, we developed analytical formulas for the probability and expected number of cancer cells with (epi)genetic instability and/or a cell growth advantage at the time of diagnosis and validated good agreement between these formulas and exact stochastic computer simulations. Third, we extended the model to consider tumor dynamics during treatment. Fourth, we confirmed that our models strongly represented clinical/experimental findings in BRCA-associated cancers. Finally, we investigated the evolutionary pathways for acquiring drug resistance during tumorigenesis before and during treatment.

We discuss the conditions for effective treatment using platinum drugs and PARPis. This study provides important implications for the evolutionary trajectories of BRCA-associated cancer progression before and during chemotherapy, depending on the growth rate, mutation rate, detection size, and treatment effects.

## Models

### Clonal expansion of two different types of mutations before diagnosis

**Figure 1:**
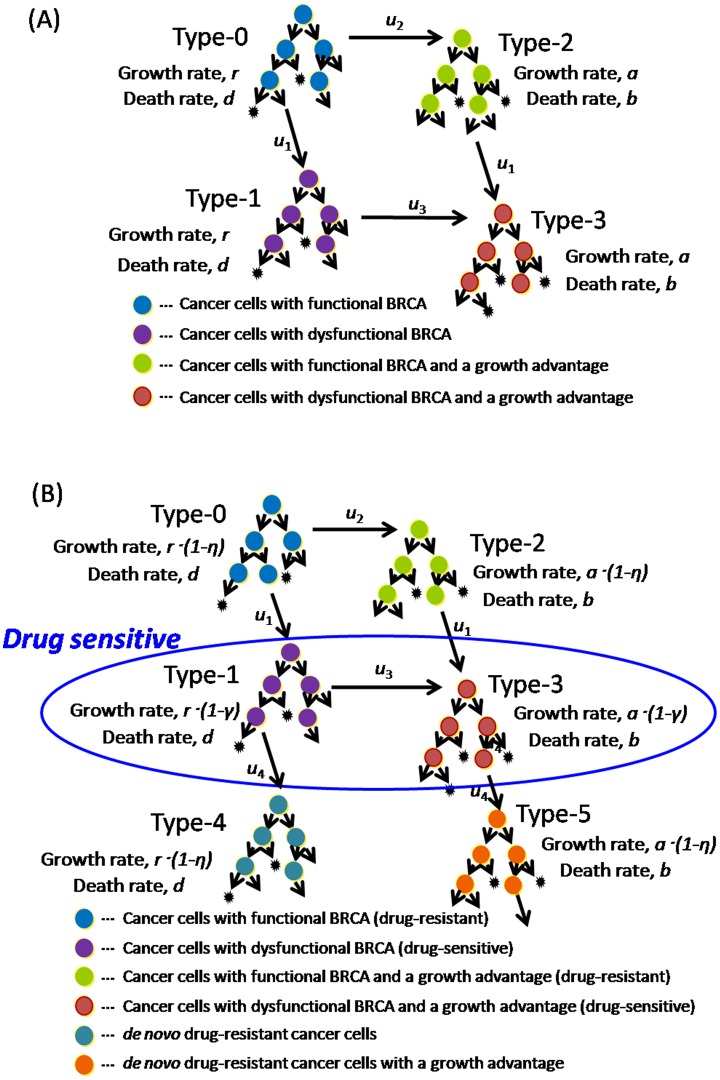
Mathematical model of BRCA-associated cancer progression (A) until diagnosis and (B) during treatment. (A) We consider an exponentially growing population of cancer cells starting from a single cell that has potential mutation targets within two genomic regions. There are two types of mutations: one facilitates (epi)genetic mutations at rate *u*_1_ and the other accelerates tumor growth at rates *u*_2_ and *u*_3_. Cancer cells with functional BRCA and an intact mutation target site for accelerated growth rates are called type-0. Cells with dysfunctional BRCA and an intact mutation target site for accelerated growth rates are called type-1. Cells carrying a mutation that accelerates uncontrollable tumor growth are called type-2 cells. Type-1 and -2 cells emerge from type-0 cells at mutation rates *u*_1_ and *u*_2_, respectively. Cells harboring both types of mutations are called type-3 cells. Type-3 cells emerge from either type-1 or -2 cells at mutation rates *u*_3_ and *u*_1_, respectively. The growth and death rates of type-0 and -1 cells are *r* and *d*, and those of type-2 and -3 cells are *a* and *b*, respectively. Once the total cell number reaches a certain size, *M*, the cancer is diagnosed. (B) To consider the situation during treatment, two populations (type-4 and -5 cells) are added to the model. Type-4 and -5 cells newly arise from type-1 and -3 cells, respectively, at rate *u*_4_ and are resistant to platinum drugs and PARPis after treatment. The growth and death rates of type-4 cells are *r* and *d*, and those of type-5 cells are *a* and *b*, respectively. The initial numbers within each type of population at diagnosis are calculated by the analytical equations derived in Eq. (S12), Eq. (S13), and Eq. (S22). We assume that neither type-4 nor -5 cells exist at the time of initial treatment. The reduced growth rates of drug-sensitive and -resistant cells caused by drug treatments are given by *γ* and *η*, respectively. Once the total cell number reaches a certain size (1.1 *M*), the cancer is considered to have relapsed.

We first describe a mathematical model of BRCA-associated cancer progression before diagnosis, considering an exponentially growing population of cancer cells derived from a single tumor-initiating cell (Fig. 1A). In this study, we assume two different types of mutations: one facilitates (epi)genetic mutations due to inactivation of BRCA function, and the other accelerates tumor growth by deregulation of the cell cycle. In BRCA-associated cancer, alterations in genes such as TP53 and PIK3CA are candidates for the latter [5].

Cancer cells with functional BRCA and an intact target for accelerating growth rate are referred to as type-0 cells. During clonal expansion, they give rise to cells harboring either of the two mutations (Fig. 1A). Cells with inactivated BRCA are type-1 cells, which have higher mutation rates than those of type-0 cells due to their error-prone DNA repair mechanisms and (epi)genetic instability. Cells carrying a mutation that accelerates uncontrollable tumor growth are type-2 cells, which grow faster than type-0 or -1 cells. Type-1 and -2 cells may give rise to cells harboring both types of mutations, referred to as type-3 cells. The term ‘mutation’ here is used collectively to include point mutations, insertions, deletions, inversions, translocations, loss of heterozygosity, and other genetic aberrations that can occur during one cell division.

Each type of population follows a continuous-time branching process. The numbers of type-0, -1, -2, and -3 cells are denoted as *w*, *x*, *y*, and *z*, respectively. We assume that the growth and death rates of type-0 are the same as those of type-1, *r* and *d*, respectively, and those of type-2 are the same as those of type-3, *a* and *b*. This assumption is based on experimental observations that inactivation of BRCA function does not have much effect on tumor growth [44]. We assume that type-2 and -3 cells have higher net growth rates than type-0 and -1 cells (*a*–*b*>*r*–*d*) since they have an additional mutation that accelerates tumor growth. The rates of mutation (i) from type-0 to -1 cells and from type-2 to -3 cells, (ii) from type-0 to -2 cells, and (iii) from type-1 to -3 cells are denoted by *u*_1_, *u*_2_, and *u*_3_, respectively.

Tumor growth begins from a single type-0 cell, *w* = 1, *x* = 0, *y* = 0, *z* = 0. In a short time period, one of the following events occurs: (i) cell division without mutation, (ii) cell division with mutation, (iii) cell death, or (iv) no transition. Tumor cells may become extinct because of stochastic fluctuations or may eventually be detected, once the total population size – the sum number of type-0, -1, -2, and -3 cells – reaches a certain size (see Materials S1 for details of the computer simulations).

### Analytical approximations

Let *P*_1_, *P*_2_, and *P*_3_ be the probabilities that type-1, -2, and -3 cells, respectively, exist when the total number of cells reaches *M*. In a previous study [32], formulas for *P*_1_, and *P*_2_ were given as
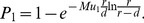
(1)

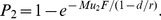
(2)


Here, 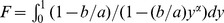 and 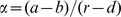.

In our model, there are two paths to the emergence of type-3 cells: through either type-1 or type-2 cells. By considering both cases independently, we derived a formula for *P*_3_ (see Materials S1 for the detailed derivation). Moreover, we consider the expected numbers of type-1, -2, and -3 cells when the total number reaches *M* to be *E*_1_, *E*_2_, and *E*_3_, respectively (see Materials S1 for the detailed derivations of these quantities).

### Emergence of resistance to platinum drugs and PARP inhibitors during treatment

Next, we considered the tumor dynamics during treatment after diagnosis. Type-0 and -2 cells are originally resistant to platinum drugs and PARPis because they can repair ICLs and DNA DSBs created by the drugs through an intact Fanconi anemia/BRCA pathway. In contrast, type-1 and -3 cells are sensitive to the drugs because of the lack of such repair mechanisms. Based on the experimental and clinical observations that secondary mutations in BRCA confer drug resistance to BRCA-deficient cells [26]–[30], we added two resistant populations, referred to as type-4 and -5 cells (Fig. 1B). Type-4 and -5 populations derive from BRCA-deficient cells (i.e., type-1 and -3 cells, respectively). We did not consider the secondary mutations from type-0 or -2 cells because they have already been defined as resistant cells. We then added two parameters as drug effects: one reduces growth rates in sensitive populations by *γ*, and the other reduces growth rates in resistant populations by *η*. In this study, we assumed that the suppression of tumor growth by drugs is achieved by a decrease in the growth rate and not by an increase in the death rate. We also assumed that treatment could decrease the growth rates of resistant cells, but at least one resistant type can increase in number even during treatment.

Based on the model described above, we investigated the cell population composition at relapse and the recurrence time intervals during treatment. We examined various combinations of treatment effects on sensitive and resistant cells, since treatment effects *in situ* have not been identified clearly and are modulated by pharmacokinetics, the tumor micro-environment, and other factors [20]. Once each parameter value is determined, the expected numbers of each population at the start of treatment can be calculated using analytical equations (Eq. (S12), Eq. (S13), and Eq. (S22)). Neither type-4 nor -5 cells exist at the time of the initial treatment. Simulations are stopped when the total number of cells exceeds 110% of the detection size, *M*, during treatment, which represents recurrence (see Materials S1 for a detailed description of the computer simulations).

## Results

### Existence probabilities and expected numbers of each cell population at diagnosis

In this section, we investigated the accuracy of the formulas for the existence probabilities as well as the expected numbers of each population at diagnosis and their dependence on each parameter. We evaluated the fit among the predictions using the formulas and the results from the stochastic computer simulations, described in Materials S1.

**Figure 2:**
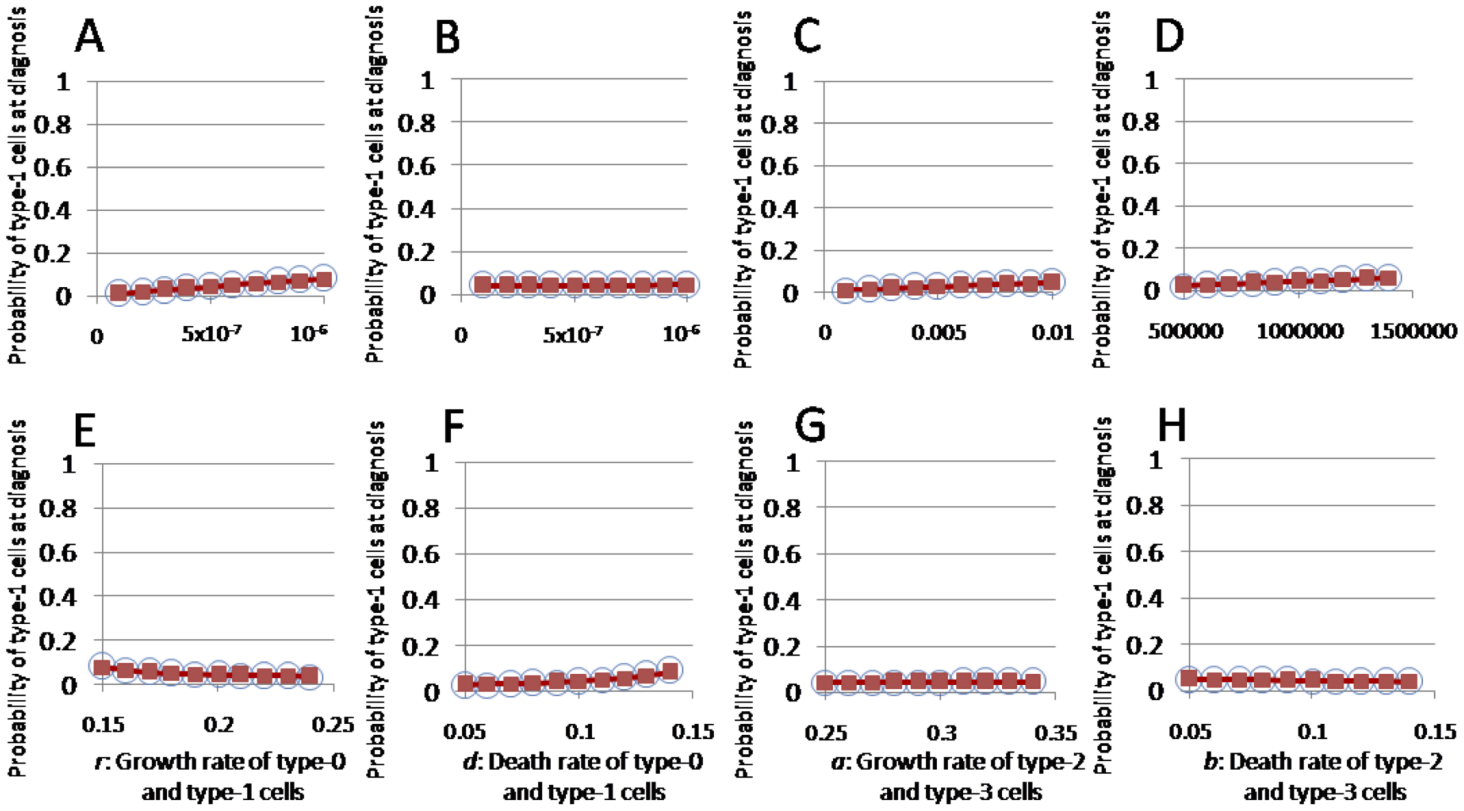
Probability of type-3 cells at diagnosis. The dependence of the probability of type-3 cell existence at diagnosis on various parameters is shown. The curves indicate the predictions of the analytical approximation, Eq. (S11), while the circles indicate the results of the direct computer simulations (system S1). Standard parameter values used in the figure are *u*_1_ = *u*_2_ = 5.0⋅10^−7^, *u*_3_ = 0.01, *M* = 10^6^, *r* = 0.2, *a* = 0.3, and *d* = *b* = 0.1.

**Figure 3:**
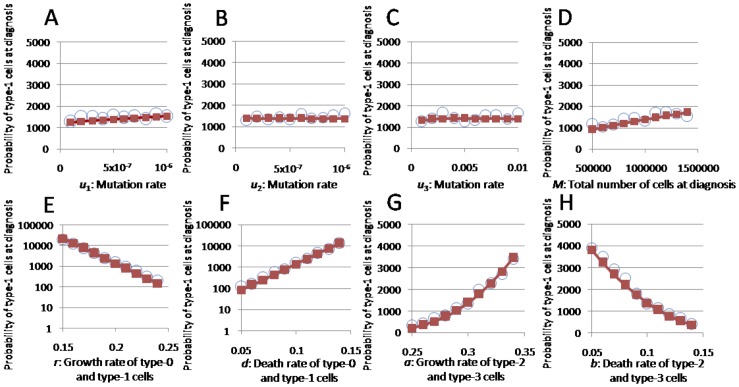
Expected numbers of type-3 cells at diagnosis. The dependence of the expected number of type-3 cells at diagnosis on various parameters is shown. The curves indicate the predictions of the analytical approximation, Eq. (S22), while the circles indicate the results of the direct computer simulations (system S1). Standard parameter values used in the figure are *u*_1_ = *u*_2_ = 5.0⋅10^−7^, *u*_3_ = 0.01, *M* = 10^6^, *r* = 0.2, *a* = 0.3, and *d* = *b* = 0.1.

First, the accuracy of the existence probability formulas and the expected numbers of type-1, -2, and -3 cells at diagnosis (Figs. 2, 3, S1-S4) were evaluated. The Eq. (1), Eq. (2), Eq. (S11), Eq. (S12), Eq. (S13), and Eq. (S22) formulas accurately predicted the results of the stochastic computer simulations. Next, we tested the accuracy of the formulas with large *u*_1_ and *u*_2_ (Figs. S5, S6). The Eq. (1), Eq. (2) and Eq. (S11) formulas accurately predicted the results of the stochastic computer simulations, with the exceptions of *P*_2_ with large *u*_1_ and *P*_1_ with large *u*_2_ (Figs. S5B, S6A). These discrepancies arose because we ignored the effects of *u*_1_ and *u*_2_ in the derivation of *P*_2_ and *P*_1_, respectively. However, when *u*_1_ or *u*_2_ is large, type-2 or -1 cells, respectively, become minor representations of the total population. Thus, this inconsistency has little effect on the expected numbers of each cell type at diagnosis (Figs. S5, S6).

We next investigated the dependence of the formulas on each parameter. The probability that type-1 cells existed at diagnosis increased as *u*_1_, *M*, and *d* increased, whereas the probability decreased as *r* increased. It was not changed by *u*_2_, *u*_3_, *a*, or *b* (Fig. S1). The probability that type-2 cells existed at diagnosis increased as *u*_2_, *M*, *d*, and *a* increased, whereas it decreased as *r* and *b* increased. It was not changed by *u*_1_ or *u*_3_ (Fig. S2). These results are consistent with those reported previously [32]. The probability that type-3 cells exist at diagnosis increased as *u*_1_, *u*_3_, *M*, and *d* increased, whereas it decreased as *r* increased. It was little changed by *u*_2_, *a*, or *b* (Fig. 2). The expected number of type-1 cells under the condition that type-1 cells existed at diagnosis increased as *u*_1_ and *M* increased, whereas it decreased as *u*_3_ increased. It was not changed greatly by *u*_2_, *r*, *d*, *a*, or *b* (Fig. S3). The expected number of type-2 cells under the condition that type-2 cells existed at diagnosis increased as *u*_2_, *M*, *d*, and *a* increased, whereas it decreased as *r* and *b* increased. It was not changed by *u*_1_ or *u*_3_ (Fig. S4). The expected number of type-3 cells under the condition that type-3 cells existed at diagnosis increased as *u*_1_, *u*_3_, *M*, *d*, and *a* increased, whereas it decreased as *r* and *b* increased. It was not changed greatly by *u*_2_ (Fig. 3).

### The proportions of the clinically significant populations at diagnosis

In this section, we investigated the following three quantities at diagnosis: (i) the proportion of cell types with high growth rates, (ii) the proportion of drug-sensitive populations, and (iii) the proportion of type-3 cells that arose from type-1 cells. The outcome of anti-tumor therapy is largely affected by the composition of a tumor at the time of therapy. For example, the proportion of cell populations with high growth rates at diagnosis reflects tumor malignancy and thus affects the control of disease by the treatment. Moreover, the proportion of drug-sensitive populations can determine the response to treatment, because platinum drugs and PARPis are effective only in BRCA-deficient cell types. Furthermore, if we specify the evolutionary pathway leading to malignant cells, it would implicate the drug-targeted cells in the prevention of tumor progression.

**Figure 4:**
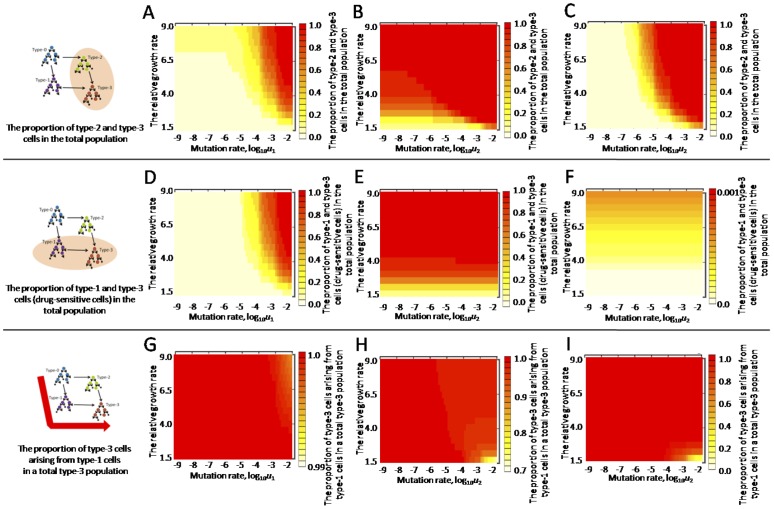
Proportion of clinically significant populations at diagnosis. (A–C) The proportion of type-2 and -3 cells with a growth advantage among the total population at diagnosis is shown over a wide range of *u*_1_, *u*_2_, and the relative growth rate of type-2 and -3 cells to that of type-0 and -1 cells is (*a*–*b*)/(*r*–*d*). (D–F) The proportion of type-1 and -3 cells (drug-sensitive cells) among the total population is shown. (G–I) The proportion of type-3 cells arising from type-1 cells among the total type-3 population is shown. Each population at diagnosis was calculated by the formulas, Eq. (S12), Eq. (S13), and Eq. (S22). Parameter values used in the figure are *u*_2_ = 10^−7^, *u*_3_ = 0.01, *M* = 10^6^, *r* = 0.2, *a* = 0.3, *d* = *b* = 0.1 (panel A, D, and G), *u*_1_ = 10^−2^ (panel B, E, and H), and *u*_1_ = 10^−7^ (panel C, F, and I).

First, we investigated the proportion of cell populations with high growth rates (i.e., type-2 and -3 cells) among the total population at diagnosis (Figs. 4A–C, S7A–B). This was calculated by dividing the sum of the expected numbers of type-2 and -3 cells by the total number, *M*. The relative growth rates of type-2 and -3 cells, compared with type-0 and -1 cells, is given by (*a*–*b*)/(*r*–*d*). The proportions of type-2 and -3 cells increased as the relative growth rate and the mutation rates (*u*_1_ and *u*_2_) increased (Figs. 4A–C, S7A–B). Second, we calculated the proportion of drug-sensitive cell populations (i.e., type-1 and -3 cells; Figs. 4D–F, S7C–D) by dividing the sum of the expected numbers of type-1 and -3 cells by the total number, *M*. The proportion increased as the relative growth rate and *u*_1_ increased (Figs. 4D, S7C–D) and, interestingly, was greatly affected by the relative growth rate and *u*_1_, but not by *u*_2_ (Figs. 4D–F, S7C–D). Third, we calculated the proportion of type-3 cells that arose from type-1 cells in a total type-3 population (Figs. 4G–I, S7E–F) by dividing the expected numbers of type-3 cells derived from type-1 cells by the expected number of type-3 cells at diagnosis. Type-3 cells emerge from type-1 cells over a wide range of parameter values except in cases where the relative growth rate is low and *u*_2_ is large (Figs. 4G–I, S7E–F). Finally, we investigated those three quantities in cases of small *u*_3_. The proportions of cell populations with high growth rates and drug sensitivity decreased in the region of large *u*_1_, and the proportion of type-3 cells that arose from type-1 cells decreased in the region of large *u*_2_ (Figs. S8, S9). The dependencies of these quantities on the relative growth rate and the mutation rates were similar to the cases of large *u*_3_ (Figs. 4, S7–S9).

### Proportion of each cell population at relapse and recurrence time intervals

In this section, we investigated the composition of each cell population in a relapsed tumor and recurrence time intervals. Two scenarios can be considered for the development of resistant populations: (i) a *de novo* resistant population arises from type-1 or -3 cells through secondary mutations during treatment and then expands, or (ii) a resistant population pre-exists in a tumor population before treatment and becomes dominant under selective pressure from the drug. The origin of the resistant population is of great importance because the treatment schedule that will best prolong the time until recurrence would be expected to differ between the two scenarios. Thus, we considered which of the two scenarios occurred preferentially over a wide range of parameter values during treatment.

**Figure 5:**
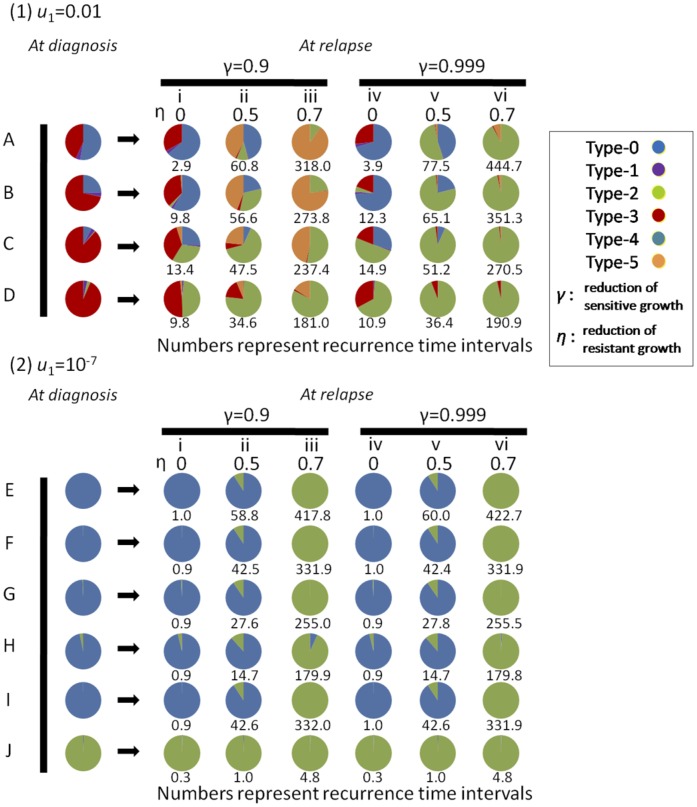
Population composition at relapse and recurrence time intervals. The population compositions at diagnosis (the time of initial treatment) and at the time of recurrence after treatment with 60 parameter sets are shown in the pie charts. The time periods until recurrence after treatment are shown as numbers under the pie charts. The time of recurrence is defined as the time point when the total number has exceeded 10% of the number at diagnosis. Each result is obtained by averaging many trials by stochastic simulations of the model under treatment (system S23). Parameter values used in the simulations, except the treatment effects, *γ* and *η*, are listed in Table 1. The letters in Table 1 correspond to those in Figure 5. Treatment effects are shown at the top of the pie charts as the reduction effects on growth rates of sensitive populations (*γ*) and those on resistant populations (*η*). We show the results separately by different values of *u*_1_; *u*_1_ is 0.01 in Figure 5(1), and 10^−7^ in Figure 5(2). The parameter values used in the figure, but not shown in Table 1 are *u*_4_ = 0.01, and *d* = *b* = 0.1.

First, we performed stochastic computer simulations of the model after diagnosis, as described in the MODELS section (Fig. 1B). We determined the composition of each cell population within a tumor at the initial time of treatment with 10 parameter combinations from the formulas Eq. (S12), Eq. (S13), and Eq. (S22) (Table 1). When *u*_1_ is large, type-3 cells become dominant (Table 1A–D). The proportion of type-3 cells becomes large as *M* increases (Table 1A–D). When *u*_1_ is small, type-0 cells become dominant (Table 1E–I), and when *u*_2_ is large, type-2 cells become dominant (Table 1J). Based on the initial tumor composition, calculated above, hundreds of stochastic simulation runs using the same initial conditions were implemented. For each parameter set listed in Table 1, we examined various drug effects on sensitive and resistant cells, *γ* and *η* (Fig. 5). The numbers of each cell type at relapse (the time when the total number reached 1.1 *M*) and the time until relapse were recorded for each run, and the averaged results are shown in Figure 5. Considering that (epi)genetic instability induced by repair pathway deficiency has a major effect on the ability to induce mutations [19], we assumed that the secondary mutation rate from type-1 and -3 cells to type-4 and -5 cells *u*_4_ was the same as *u*_3_.

**Table 1:** Parameter sets used for the analysis in Figure 5 and the expected numbers of cells at diagnosis.

(1) *u*_1_ = 0.01	Parameter values				Expected Number (Proportion) of Cells at Diagnosis			
	*a*	*u*_2_	*u*_3_	*M*	Type-0	Type-1	Type-2	Type-3
A	0.4	10^−7^	10^−2^	10^5^	52843 (0.53)	3885 (0.03)	31 (0.00)	43249 (0.43)
B				10^6^	245500 (0.25)	37102 (0.04)	1446 (0.00)	715950 (0.72)
C				10^7^	750925 (0.08)	354324 (0.04)	66724 (0.01)	8828026 (0.88)
D				10^8^	0 (0.00)	3383775 (0.03)	3014563 (0.03)	93789333 (0.94)

(2) *u*_1_ = 10^−7^	Parameter values				Expected Number (Proportion) of Cells at Diagnosis			
	*a*	*u*_2_	*u*_3_	*M*	Type-0	Type-1	Type-2	Type-3
E	0.4	10^−7^	10^−2^	10^5^	99967 (1.00)	0 (0.00)	31 (0.00)	2 (0.00)
F				10^6^	998468 (1.00)	2 (0.00)	1446 (0.00)	82 (0.00)
G				10^7^	9929430 (0.99)	17 (0.00)	66738 (0.01)	3813 (0.00)
H				10^8^	96809333 (0.97)	166 (0.00)	3014563 (0.03)	175936 (0.00)
I			10^−4^	10^6^	998549 (1.00)	2 (0.00)	1446 (0.00)	2 (0.00)
J		10^−2^	10^−2^	10^6^	6149 (0.01)	2 (0.00)	993765 (0.99)	83 (0.00)

The expected numbers of type-1, -2, and -3 cells at diagnosis were calculated using Eq. (S12), Eq. (S13), and Eq. (S22). The remainder of the total number is considered to comprise the number of type-0 cells. The proportion of each type is shown in parentheses. Parameter values used in Figure 5 are *r* = 0.2 and *d* = *b* = 0.1.

We then investigated the proportion of each cell population at the time of recurrence with large *u*_1_ (Fig. 5). The proportion of type-3 cells in a tumor population at relapse is large when *η*is zero but decreases as *η* becomes large (Fig. 5A–D). In the former case, resistant cells grow too rapidly for type-3 cells to disappear until tumor relapse; however, in the latter case, slow growth of resistant cells facilitates mutational events from type-3 to -5 cells and also confers a time of elimination by negative selection on type-3 cells. Type-5 cells have more chance of being present at the time of relapse in the latter condition (Fig. 5A–D). The proportion of type-2 cells increases as *M*, *γ*, and *η*increase (Fig. 5A–D). When the tumor size at diagnosis, *M*, is large, a large proportion of type-2 cells is likely to be present (Table 1), resulting in a large proportion of type-2 cells at relapse. Type-1 and -4 cells are hardly detected at relapse because they are rarely present at the time of initial treatment and grow slower than type-3 and -5 cells.

Furthermore, we investigated the proportion of each cell population at the time of recurrence with small *u*_1_ (Fig. 5). With all treatment effect combinations, type-0 or -2 cells became dominant (Fig. 5E–I), because they were likely to be present in large proportions at diagnosis (Table 1E–J) and are resistant to the drugs. The proportion of type-2 cells at relapse became large when *η* and *u*_2_ were large (Fig. 5E–J).

Finally, we investigated the length of time between diagnosis and recurrence to find the cases in which platinum drugs and PARPis effectively prolong the time until recurrence. The time until recurrence increased as *η* increased, whereas it was not changed greatly by *γ* (Fig. 5). Interestingly, in the case of large *u*_1_ ( = 10^−2^ *)* and small *η* ( = 0), the time until recurrence became longer than that with small *u*_1_ ( = 10^−7^ *)* or small *η* ( = 0; Fig. 5), because the proportion of drug-sensitive cells becomes dominant at the time of initial treatment when *u*_1_ is large (Table 1A–D). Additionally, we confirmed the robustness of the results over a wide range of *u*_1_ and *u*_2_ (Table S1, Fig. S10).

### Agreement between the results and clinical evidence

Next, we investigated whether our models described clinical and experimental observations in BRCA-associated cancers (Table 2). We categorized BRCA-associated cancers into two types in terms of different inactivation rates of BRCA, *u*_1_. BRCA inactivation is induced by LOH in ovarian cancer [7], [12] and basal-like breast cancer [5], [6], indicating high mutation rates of *u*1. We assume that in this case, *u*_1_ is 10^−2^ at maximum. This value is based on the observation of an inactivated second allele in cells with (epi)genetic instability [45]. In contrast, particularly in breast cancer, biallelic loss of BRCA is not commonly observed at diagnosis, suggesting LOH is not involved in the mutational event [9]. We assume that in this case, *u*_1_ is 10^−7^. The rate of mutation without LOH has been determined experimentally by investigating DNA replication fidelity [46]. Here, the mutation rate from type-1 to type-3 cells, *u*_3_, is assumed to be as high as the mutation rate induced by LOH, because we took into account the additive (epi)genetic instability effect caused by BRCA inactivation [45]. Additionally, when we considered the agreement between our results and the clinical evidence, we focused on the small treatment effects in resistant populations. This assumption seems reasonable because recurrence during treatment does, in fact, occur in the clinic, and the small effects of platinum drugs and PARPis on resistant cells have been confirmed experimentally [27], [29], [30], [47].

**Table 2:** Agreements between the results and the clinical and molecular evidence.

Results	Figures and Tables	Clinical and molecular evidence	References
(1)Cases of high mutation rate (*u*_1_ = 0.01)			
Coexistence of multiple populations at diagnosis and relapse	Figs. 4, 5, S7, S9, S10, Table 1	Intra-tumor genetic heterogeneity in ovarian and breast cancer	48,49
High frequency of cells with a growth advantage at diagnosis	Figs. 4, S7	Observation of TP53 mutation in more than 90% of ovarian cancers	5,7,50
		Observation of TP53 mutation in more than 80% of basal-like breast cancers	5
High frequency of BRCA-inactivated (drug-sensitive) cells at diagnosis	Figs. 4D, 4E, S7C, S7D	High frequency of (epi)genetic instability in high grade serous ovarian cancer	51,52,53
Ten-fold survival benefit in treatment with platinum drugs and PARPis	Fig. 5A(i)–D(i), E(i)–H(i)	Good prognosis by the platinum-based and PARPis chemotherapy in patients with BRCA mutation.	7,22,23,62
High frequency of drug-sensitive type-3 cells at relapse	Figs. 5A(i)–D(i), A(iv)–D(iv), S10(i), S10(iv)	Some patients can be re-treated with the same drugs which previously showed good sensitivity	54
		Good responses by PARPis to platinum-sensitive relapsed tumors	55
Proportion of type-2 cells increases as tumor detection size increases	Fig. 5A–D, Table 1	Detection in early stage is favorable for overall survival in ovarian cancer	56
(2) Cases of low mutation rate (*u*_1_ = 10^−7^)			
Wide range of aggressiveness at diagnosis	Figs. 4A, 4C, S7A, S7B	Observation of TP53 mutation in 12% to 29% in luminal breast cancers	5
Low frequency of BRCA-inactivated (drug-sensitive) cells at diagnosis	Figs. 4D, 4F, S7C, S7D	Late BRCA1 inactivation in BRCA1-associated breast tumors	11
Early treatment failure due to pre-existing drug-resistant cells	Figs. 5E–J, S10 (i), S10(iv)	Small effects by DNA damaging drugs in BRCA-associated breast cancers	57

First, we considered the high mutation rate cases, *u*_1_ = 10^−2^, such as ovarian cancer and basal-like breast cancer, in which BRCA inactivation is frequently observed during tumorigenesis (Table 2). In most computer simulation cases, mixed populations coexisted at both diagnosis and relapse, indicating heterogeneity in the tumor (Figs. 4, 5, S7, S8, S9, S10, and Tables 1 and S1). These results are consistent with clinical observations of intra-tumoral genetic heterogeneity in ovarian and breast cancers [48], [49]. When we focused on the aggressiveness of the tumor, the majority of tumor cells at diagnosis had a high proliferation rate under large *u*_1_ conditions (Figs. 4A, 4B, S7A, S7B). The results are consistent with recent reports that TP53 mutations are observed in more than 90% of ovarian cancers [5], [7], [50] and 80% of basal-like breast cancers [5]. BRCA function is inactivated in a large proportion of tumors at diagnosis with large *u*_1_ (Figs. 4D, 4E, S7C, S7D). Thus, the tumor cells are expected to show (epi)genetic instability and sensitivity to platinum drugs and PARPis while exhibiting a high proliferation rate over a wide range of parameter values (Fig. 4B, 4E). These results fit with the reports of high (epi)genetic instability frequency in high-grade serous ovarian cancer [51], [52], [53].

Moreover, our results indicate that when there is no treatment effect on resistant cancer cells, platinum drugs and PARPis have a ∼10-fold survival benefit for cancers with large *u*_1_ compared with the cases with small *u*_1_ (Fig. 5A(i)–D(i), (i)–H(i)). This observation may explain why clinical cases of ovarian cancer with BRCA mutations show a better prognosis than those with wild-type BRCA during chemotherapy [7], [22], [23]. Our results support the conventional first-line regimen of platinum-based chemotherapy in BRCA-associated ovarian cancer.

Furthermore, in the case of no treatment effect in resistant cancer cells, drug-sensitive type-3 cells still exist in a large proportion of tumors at relapse (Figs. 5A(i)–D(i), 5A(iv)–D(iv), S10(i), S10(iv)). The results indicate that retreatment with platinum drugs and PARPis after recurrence may still be worthy of consideration. Indeed, this is supported by a recent report in which re-treatment with cisplatin was effective in patients who exhibited good sensitivity to platinum drugs with prior treatment [54], and that PARPis were effective in platinum-sensitive relapsed tumors [55]. Notably, tumors also contain resistant cells in such a situation, and recurrence is inevitable if we simply continue the same strategy as before (Figs. 5A(i)–D(i), A(iv)–D(iv), S10(i), S10(iv)).

Finally, we observed that the proportion of type-2 cells at relapse increased as the total cell number at diagnosis increased (Fig. 5A–D, Table 1). This causes early treatment failure due to the pre-existence of resistant cells. This result is consistent with the clinical evidence that early-stage and smaller residual tumor volumes were favorable characteristics for overall survival in ovarian cancer [56]. Taken together, the model could reproduce the clinical evidence in cases of frequent BRCA inactivation during tumorigenesis, such as in ovarian cancer or basal-like breast cancer.

We also considered the case of a low mutation rate, *u*_1_ = 10^−7^. This represents tumorigenesis in non-basal-like breast cancer with BRCA heterozygotes [10]. Regarding the aggressiveness of the tumor, the results with small *u*_1_ were similar to clinical observations in luminal breast cancer, in which a wide range of aggressiveness occurs (Figs. 4A, 4C, S7A, S7B). Indeed, the prediction may explain the report that TP53 mutations are observed in 12–29% of luminal breast cancers, much lower than that in ovarian or basal-like breast cancers [5]. In marked contrast to the situation with a high mutation rate, *u*_1_ = 10^−2^, the proportion of cells sensitive to platinum drugs and PARPis is very small (<0.002) at diagnosis over the global range of parameter values (Figs. 4D, 4F, S7C, S7D). These results agree well with the finding that TP53 mutations occur first, instead of BRCA1 inactivation, in evolutionary pathways of BRCA1-associated luminal breast tumors [11]. Tumors dominated by resistant populations at diagnosis result in early treatment failure when there is no treatment effect on resistant cells (Figs. 5E–J, S10(i), S10(iv)). These results are consistent with current therapeutic outcomes for BRCA-associated breast cancers, in which DNA-damaging drugs did not show substantial effects with a single agent [57]. These results may also explain why clinical trials on PARPis treatment in patients with triple-negative breast cancer failed, with patients showing no response, in contrast to substantial responses of BRCA-associated ovarian cancers in the same trial [58], because the patient populations with triple-negative breast cancer contained not only basal-like but also non-basal breast cancers [59]. In summary, we have been able to reproduce BRCA-associated cancer types, such as non basal-like breast cancer, in which BRCA inactivation is not frequently observed during tumorigenesis.

## Discussion

In this study, we developed mathematical models of BRCA-associated cancer progression before and during treatment with platinum drugs or PARPis. Next, we investigated the frequency of each cell population at diagnosis and the evolution of drug resistance during treatment. We derived analytical approximations for the proportions of drug-sensitive and -resistant cells at diagnosis and explored evolutionary pathways involved in acquiring drug resistance. Recurrence-free intervals were also investigated over a wide parameter range. Moreover, we reproduced clinical/experimental observations successfully. In the parameter settings for ovarian or basal-like breast cancer, in which BRCA inactivation is commonly observed during tumorigenesis, the models succeeded in capturing the following clinical evidence: (i) high tumor heterogeneity and high prevalence of aggressive tumor cells, (ii) high frequency of (epi)genetic instability and sensitivity to platinum drugs and PARPis, (iii) long recurrence-free intervals due to a high frequency of BRCA-mutated cell populations, (iv) possibility of re-treatment with platinum drugs and PARPis, and (v) early treatment failure in the case of large detection size. Moreover, we have reproduced the clinical evidence that a therapy reliant on DNA repair deficiency would not be a promising approach in the case of non-basal-like breast cancer, in which BRCA heterozygosity is sometimes retained during tumorigenesis.

It is clinically important to reveal the trajectory of drug resistance development during the treatment of ovarian carcinoma [60]. In this study, we investigated two possibilities for the evolution of drug resistance in a large *u*_1_ condition: (i) expansion of a pre-existing intrinsic sub-population and (ii) acquisition of drug resistance due to secondary mutations during treatment. When there are 90% growth reducing effects on sensitive cells and no effect on resistant cells, the majority of resistant cells are pre-existing resistant tumor cells, such as type-0 or -2 cells, in a relapsed tumor (Fig. 5A(i)–D(i)). However, when treatment effects on resistant cells are not small, resistant cells may emerge due to secondary mutations in BRCA during treatment (Fig. 5A(iii)–D(iii)). In this case, it takes a long time for *de novo* resistant cells in a relapsed tumor to emerge and become dominant (Fig. 5A(ii), 5A(iii), 5B(ii), 5B(iii), 5C(iii)). Given that large treatment effects on resistant cells in the clinic are less plausible, we suggest that drug resistance acquired by novel mutations during treatment occurs in only a small proportion of patients after long-term exposure to chemotherapy. Indeed, only 6.3% (4/64 patients) of ovarian cancer patients showed *de novo* resistance to treatment [29], and the secondary mutations did not become detectable until more than a decade after chemotherapy for ovarian and breast cancers [29], [30]. When there was more than 99% tumor growth reduction on sensitive cells, most of the cells in a relapsed tumor were originally present at the start of therapy (Fig. 5A–D). Collectively, these results indicate that the origin of resistant cells in a relapsed tumor can vary according to the tumor characteristics and treatment effects on both sensitive and resistant cells.

**Figure 6:**
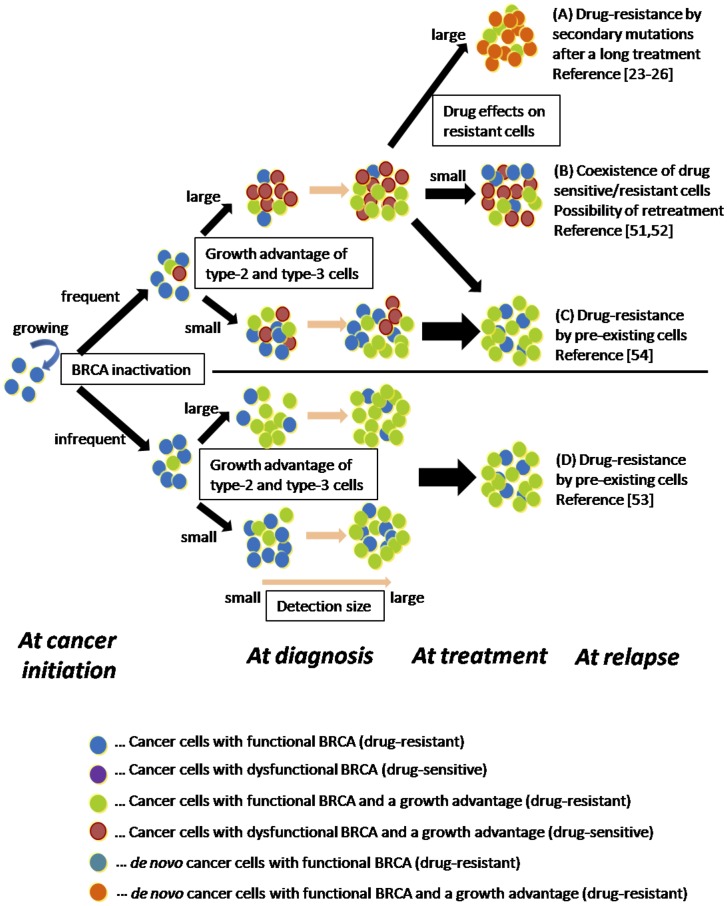
Schematic illustration of evolutionary trajectories of BRCA-associated cancers. The hypothesis on the evolutionary trajectories of BRCA-associated cancers is illustrated. Treatment outcomes are determined by the following four conditions: (i) the frequency of BRCA inactivation, (ii) growth advantage of type-2 and -3 cells, (iii) the detection size, and (iv) drug effects on resistant cells. (A) Drug resistance by secondary mutations emerges after long-term treatment in the case of (i) as high, (ii) as large, (iii) as large, and (iv) as large. (B) Drug-sensitive and -resistant cells coexist at relapse and retreatment with the same drugs is conceivable in the case of (i) as high, (ii) as large, (iii) as not large, and (iv) as small. (C and D) Drug resistance due to pre-existing resistant cells emerges in the case of (i) as high, (ii) as small, (iii) as large and (iv) as any, or in the case of (i) as small. The reference numbers indicate clinical observations, each of which corresponds to each outcome in the evolutionary trajectories.

Based on the results from the models, our hypothesis on the evolutionary trajectories of BRCA-associated cancers is illustrated in Figure 6. The frequency of BRCA inactivation is a major determinant in the future sensitivity to platinum drugs and PARPis. If it is high, the drug-sensitive tumor population appears at diagnosis. In this situation, drug-sensitive and -resistant cells may coexist at relapse after treatment, and effective retreatment is conceivable (Fig. 6B). Populations with secondary mutations may emerge after long-term treatment if the drugs effectively suppress the growth of resistant cells as well as sensitive cells (Fig. 6A). If a sufficient growth advantage is not obtained by the mutation to accelerate tumor growth or the tumor is diagnosed at a late phase, the tumor tends to contain drug-resistant cells at the start of therapy (Fig. 6C). If BRCA inactivation does not occur frequently during tumorigenesis, the tumor will be resistant to platinum drugs and PARPis at the start of therapy (Fig. 6D). Taken together, platinum drugs and PARPis should be effective if (i) BRCA inactivation occurs, (ii) treatment is started early, and (iii) tumor growth is rapid. Our results may help determine individualized treatment options for patients with BRCA-associated cancers in the clinic.

This research provides theoretical insights into the therapeutic approaches for different types of cancers that share major mutations in tumorigenesis. Our results suggest that platinum drug and PARPi treatment strategies dependent on the underlying defects in DNA repair mechanisms should be commonly effective in cancers in which BRCA inactivation occurs frequently, such as basal-like breast and ovarian cancers (Fig. 5A–D), as reported [60]–[62]. In fact, basal-like breast cancer shares molecular features with serous ovarian cancer, but not with other breast cancers [5]. In ovarian cancer, the time interval from completion of platinum-based chemotherapy to disease progression has been conventionally used as an indicator to predict the response to subsequent treatment with platinum-containing regimens [63]. However, our results indicate that the cell population profiles in BRCA-associated cancers at relapse vary largely in accordance with the treatment effects in sensitive and resistant populations (Fig. 5). Thus, assessment of whether the relapsed tumor cells restore HR function, for example, using DNA sequencing and Rad51 foci formation assays, could be key to determining the sequential treatment strategies.

Another important result is the identification of the evolutionary paths leading to tumor cells with malignant characteristics. Interestingly, type-3 cells, which have high mutation and growth rates, emerged exclusively from type-1, and not type-2, cells with high mutation rates regardless of *u*_1_, *u*_2_, or the relative growth rate, (*a*–*b*)/(*r*–*d*) (Figs. 4G–I, S7E, S7F). These results suggest that even though mutation rates enhanced by dysfunction in DNA repair mechanisms itself do not confer a growth advantage to cells, they subsequently induce additional mutations, some of which may accelerate tumor growth. In this case, the pervasive characteristics of tumor cells regarding dysfunctional BRCA provide a therapeutic opportunity, as seen with platinum drugs and PARPis.

In this study, we considered that tumor cells grow independently during tumorigenesis. In the initial progression of a carcinoma, exponential growth without tumor competition is often assumed. Thus, we adopted the simple growth mode without density effects in the first study regarding the evolution of drug resistance to platinum drugs and PARPis in BRCA-associated cancers. We also considered a simple but feasible dosing strategy of continuous drug administration and assumed the secondary mutation rate *u*_4_ to be the same as *u*_3_. This assumption is based on evidence that (epi)genetic instability induced by deficiency in the repair pathway has a major effect on determining the ability to induce mutations [19]. Moreover, we did not distinguish the treatment effects on several types of resistant cells. Instead, we examined possible situations during treatment over a wide range by considering various parameter combinations of treatment effects on both sensitive and resistant cells. Furthermore, we assumed that secondary mutations before treatment were very rare and that type-4 and -5 populations were introduced only after treatment. This is supported by the evidence that secondary mutations are observed experimentally only when platinum drugs and PARPis are administered to cells [27], [28]. Because the effects of inactivation on DNA repair mechanisms vary between BRCA1 and BRCA2, the mutation rate from type-1 to -3 cells, *u*3, can be low [10]. We then investigated the proportions of cell populations at diagnosis with a small *u*3 (Figs. S8, S9). Although parameter dependence shows a similar pattern to that with a large *u*_3_ (Figs. 4, S7, S8, S9), the populations with high growth rates and drug sensitivity (type-3) are less prevalent under these conditions. This result agrees in part with those of clinical cases, in which platinum-resistant cancer recurs in ∼25% of ovarian patients within 6 months [7]. These results indicate that, in general, *u*_3_ may not be small [45].

Even though we have focused on BRCA1/2 inactivation in impaired HR function, the frequency of BRCA1/2 mutations does not explain all cases of HR impairment in BRCA-associated cancers. Indeed, 10–20% of ovarian cancers [7] and ∼20% of breast cancers show inactivation of BRCA1/2 [5]. However, The Cancer Genome Atlas analysis of serous ovarian cancer documented that ∼50% of serous ovarian cases [64] and 20% of triple-negative breast cancers [65] might have disrupted HR repair mechanism(s). Recently, many molecular mutations have been identified that confer BRCA-like characteristics to tumors, such as the Fanconi anemia protein family [66] and PTEN [67]. Thus, there is broad recognition of HR dysfunction in tumorigenesis. Our models can be interpreted as type-1 populations include cells not only with inactivated BRCA1/2 but also with ‘BRCAness’, that is, HR defects without mutations in BRCA1/2.

Collectively, we considered the evolutionary dynamics of BRCA-associated cancer before and during treatment and identified effective treatment conditions using platinum drugs and PARPis in agreement with clinical and experimental observations. These results may be applied to other BRCA-associated cancers, such as prostate, pancreatic, and uterine serous. The frequency of patients with BRCA mutations is small in these cancers. However, our results indicate that treatment with platinum drugs and PARPis in specific patients with HR impairment due to BRCA mutations might be an effective option essentially as a tailor-made therapy. Furthermore, our method in which we considered different types of cancers (ovarian and breast) in the same model according to the representative mutational status can be applied to other cancers if the major milestones during tumorigenesis are shared among these cancers. This approach will provide novel insights into therapeutic strategies from the viewpoint of pathway-targeted therapy against multiple cancers.

## Supporting Information

Figure S1 **The probability of type-1 cells at diagnosis.** The figure shows the dependence of the probability of the existence of type-1 cells at diagnosis on various parameters. The curves indicate the predictions of the analytical approximation, Eq. (1), while the circles indicate the results of the direct computer simulation (system S1). Standard parameter values used in the figure are *u*_1_ = *u*_2_ = 5.0⋅10^−7^, *u*_3_ = 0.01, *M* = 10^6^, *r* = 0.2, *a* = 0.3, *d* = *b* = 0.1.
(TIF)

Figure S2 **The probability of type-2 cells at diagnosis.** The figure shows the dependence of the probability of the existence of type-2 cells at diagnosis on various parameters. The curves indicate the predictions of the analytical approximation, Eq. (2), while the circles indicate the results of the direct computer simulation (system S1). Standard parameter values used in the figure are *u*_1_ = *u*_2_ = 5.0⋅10^−7^, *u*_3_ = 0.01, *M* = 10^6^, *r* = 0.2, *a* = 0.3, *d* = *b* = 0.1.
(TIF)

Figure S3 **The expected number of type-1 cells at diagnosis.** The figure shows the dependence of the expected number of type-1 cells at diagnosis on various parameters. The curves indicate the predictions of the analytical approximation, Eq. (S12), while the circles indicate the results of the direct computer simulation (system S1). Standard parameter values used in the figure are *u*_1_ = *u*_2_ = 5.0⋅10^−7^, *u*_3_ = 0.01, *M* = 10^6^, *r* = 0.2, *a* = 0.3, *d* = *b* = 0.1.
(TIF)

Figure S4 **The expected number of type-2 cells at diagnosis.** The figure shows the dependence of the expected number of type-2 cells at diagnosis on various parameters. The curves indicate the predictions of the analytical approximation, Eq. (S13), while the circles indicate the results of the direct computer simulation (system S1). Standard parameter values used in the figure are *u*_1_ = *u*_2_ = 5.0⋅10^−7^, *u*_3_ = 0.01, *M* = 10^6^, *r* = 0.2, *a* = 0.3, *d* = *b* = 0.1.
(TIF)

Figure S5 **The probabilities and the expected numbers of each population at diagnosis with large** ***u*** **_1_.** The figure shows the probabilities of the existence of type-1, -2, and -3 cells and the expected numbers of type-1, -2, and -3 cells at diagnosis in a region of large *u*_1_. The curves indicate the predictions of the analytical approximations, Eq. (1), Eq. (2), Eq. (S11), Eq. (S12), Eq. (S13), and Eq. (S22), while the circles indicate the results of the direct computer simulations (system S1). Parameter values used in the figure are *u*_2_ = 5.0⋅10^−7^, *u*_3_ = 0.01, *M* = 10^6^, *r* = 0.2, *a* = 0.3, *d* = *b* = 0.1.
(TIF)

Figure S6 **The probabilities and the expected numbers of each population at diagnosis with large** ***u*** **_2_.** The figure shows the probabilities of the existence of type-1, -2, and -3 cells and the expected numbers of type-1, -2, and -3 cells at diagnosis in a region of large *u*_2_. The curves indicate the predictions of the analytical approximations, Eq. (1), Eq. (2), Eq. (S11), Eq. (S12), Eq. (S13), and Eq. (S22), while the circles indicate the results of the direct computer simulations (system S1). Parameter values used in the figure are *u*_1_ = 5.0⋅10^−7^, *u*_3_ = 0.01, *M* = 10^6^, *r* = 0.2, *a* = 0.3, *d* = *b* = 0.1.
(TIF)

Figure S7 **Proportion of clinically significant populations at diagnosis.** (A–B) The proportion of type-2 and -3 cells with a growth advantage in the total population at diagnosis is shown in a wide region of *u*_1_, *u*_2_. (C-D) The proportion of type-1 and -3 cells (drug-sensitive cells) in the total population is shown. (E–F) The proportion of type-3 cells arising from type-1 cells in a total type-3 population is shown. Each population at diagnosis is calculated by the formulas, Eq. (S12), Eq. (S13), and Eq. (S22). Parameter values used in the figure are *u*_3_ = 10^−2^, *M* = 10^6^, *r* = 0.2, *a* = 0.3, *d* = *b* = 0.1 (panel A, C, and E); and *a* = 0.6 (panel B, D, and F).
(TIF)

Figure S8 **Proportion of clinically significant populations at diagnosis with a low mutation rate,** ***u*** **_3_.** (A–C) The proportion of type-2 and -3 cells with a growth advantage in the total population at diagnosis is shown in a wide region of *u*_1_, *u*_2_ and the relative growth rate of type-2 and -3 cells to that of type-0 and -1 cells, (*a*–*b*)/(*r*–*d*). (D–F) The proportion of type-1 and -3 cells (drug-sensitive cells) in the total population is shown. (G–I) The proportion of type-3 cells arising from type-1 cells in a total type-3 population is shown. Each population at diagnosis is calculated by the formulas, Eq. (S12), Eq. (S13), and Eq. (S22). Parameter values used in the figure are *u*_2_ = 10^−7^, *u*_3_ = 10^−4^, *M* = 10^6^, *r* = 0.2, *a* = 0.3, *d* = *b* = 0.1 (panel A, D, and G); *u*_1_ = 10^−2^ (panel B, E, and H); and *u*_1_ = 10^−7^ (panel C, F, and I).
(TIF)

Figure S9 **Proportion of clinically significant populations at diagnosis with a low mutation rate,** ***u*** **_3_.** (A–B) The proportion of type-2 and -3 cells with a growth advantage in the total population at diagnosis is shown in a wide region of *u*_1_, *u*_2_. (C–D) The proportion of type-1 and -3 cells (drug-sensitive cells) in the total population is shown. (E–F) The proportion of type-3 cells arising from type-1 cells in a total type-3 population is shown. Each population at diagnosis is calculated by the formulas, Eq. (S12), Eq. (S13), and Eq. (S22). Parameter values used in the figure are *u*_3_ = 10^−4^, *M* = 10^6^, *r* = 0.2, *a* = 0.3, *d* = *b* = 0.1 (panel A, C, and E); and *a* = 0.6 (panel B, D, and F).
(TIF)

Figure S10 **The population composition at relapse and recurrence time intervals in a wide region of** ***u*** **_1_ and** ***u*** **_2_.** The population compositions at diagnosis (the initial time of treatment) and at the time of recurrence after treatment in a wide region of *u*_1_ and *u*_2_ are shown in pie charts. The time periods until the time of recurrence after treatment are shown as numbers under the pie charts. The time of recurrence is defined as the time point when the total number reaches 10% larger than the number at diagnosis. Each result is obtained by averaging a lot of trials by stochastic simulations of the model under treatment (system S23). The parameter values used in the figure except *u*_1_ and *u*_2_ are *u*_3_ = *u*_4_ = 0.01, *M* = 10^6^, *a* = 0.4, and *d* = *b* = 0.1. Treatment effects are shown at the top of pie charts as the reduction effects on growth rates of sensitive populations (*γ*) and those on resistant populations (*η*).
(TIF)

Materials S1 **Supplementary material.**
(DOCX)

Table S1 **Parameter sets used for the analysis in Supplementary Figure S10 and expected numbers of cells at diagnosis.** The expected numbers of type-1, -2, and -3 cells at diagnosis are calculated by Eq. (S12), Eq. (S13), and Eq. (S22). The number of type-0 cells is considered as the rest of the total number. The proportion of each type is shown in parentheses. The cases where the sum of type-0, type-1, type-2, and type-3 cells exceeded *M* were excluded. Parameter values used in the figure are *u*_3_ = 0.01, *M* = 10^6^, *r* = 0.2, *a* = 0.4, *d* = *b* = 0.1.
(EPS)

## References

[1] Fackenthal JD, Olopade OI (2007) Breast cancer risk associated with BRCA1 and BRCA2 in diverse populations. Nat Rev Cancer 7: 937–948. 18034184 10.1038/nrc2054

[2] Castro E, Goh C, Olmos D, Saunders E, Leongamornlert D, et al (2013) Germline BRCA mutations are associated with higher risk of nodal involvement, distant metastasis, and poor survival outcomes in prostate cancer. J Clin Oncol 31: 1748–1757. 23569316 10.1200/JCO.2012.43.1882 PMC3641696

[3] Skoulidis F, Cassidy LD, Pisupati V, Jonasson JG, Bjarnason H, et al (2010) Germline Brca2 heterozygosity promotes Kras(G12D) -driven carcinogenesis in a murine model of familial pancreatic cancer. Cancer Cell 18: 499–509. 21056012 10.1016/j.ccr.2010.10.015

[4] Pennington KP, Walsh T, Lee M, Pennil C, Novetsky AP, et al (2013) BRCA1, TP53, and CHEK2 germline mutations in uterine serous carcinoma. Cancer 119: 332–338. 22811390 10.1002/cncr.27720 PMC3966566

[5] Cancer Genome Atlas N (2012) Comprehensive molecular portraits of human breast tumours. Nature 490: 61–70. 23000897 10.1038/nature11412 PMC3465532

[6] Greenup R, Buchanan A, Lorizio W, Rhoads K, Chan S, et al (2013) Prevalence of BRCA mutations among women with triple-negative breast cancer (TNBC) in a genetic counseling cohort. Ann Surg Oncol 20: 3254–3258. 23975317 10.1245/s10434-013-3205-1

[7] Cancer Genome Atlas Research N (2011) Integrated genomic analyses of ovarian carcinoma. Nature 474: 609–615. 21720365 10.1038/nature10166 PMC3163504

[8] Buchholz TA, Wu X, Hussain A, Tucker SL, Mills GB, et al (2002) Evidence of haplotype insufficiency in human cells containing a germline mutation in BRCA1 or BRCA2. Int J Cancer 97: 557–561. 11807777 10.1002/ijc.10109

[9] Rennstam K, Ringberg A, Cunliffe HE, Olsson H, Landberg G, et al (2010) Genomic alterations in histopathologically normal breast tissue from BRCA1 mutation carriers may be caused by BRCA1 haploinsufficiency. Genes Chromosomes Cancer 49: 78–90. 19839046 10.1002/gcc.20723

[10] Roy R, Chun J, Powell SN (2012) BRCA1 and BRCA2: different roles in a common pathway of genome protection. Nat Rev Cancer 12: 68–78. 10.1038/nrc3181 PMC4972490 22193408

[11] Martin RW, Orelli BJ, Yamazoe M, Minn AJ, Takeda S, et al (2007) RAD51 up-regulation bypasses BRCA1 function and is a common feature of BRCA1-deficient breast tumors. Cancer Res 67: 9658–9665. 17942895 10.1158/0008-5472.CAN-07-0290

[12] King TA, Li W, Brogi E, Yee CJ, Gemignani ML, et al (2007) Heterogenic loss of the wild-type BRCA allele in human breast tumorigenesis. Ann Surg Oncol 14: 2510–2518. 17597348 10.1245/s10434-007-9372-1

[13] Mullany LK, Fan HY, Liu Z, White LD, Marshall A, et al (2011) Molecular and functional characteristics of ovarian surface epithelial cells transformed by KrasG12D and loss of Pten in a mouse model in vivo. Oncogene 30: 3522–3536. 21423204 10.1038/onc.2011.70 PMC3139785

[14] Wu R, Baker SJ, Hu TC, Norman KM, Fearon ER, et al (2013) Type I to type II ovarian carcinoma progression: mutant Trp53 or Pik3ca confers a more aggressive tumor phenotype in a mouse model of ovarian cancer. Am J Pathol 182: 1391–1399. 23499052 10.1016/j.ajpath.2012.12.031 PMC3620412

[15] D'Andrea AD, Grompe M (2003) The Fanconi anaemia/BRCA pathway. Nat Rev Cancer 3: 23–34. 12509764 10.1038/nrc970

[16] Patel KJ, Yu VP, Lee H, Corcoran A, Thistlethwaite FC, et al (1998) Involvement of Brca2 in DNA repair. Mol Cell 1: 347–357. 9660919 10.1016/s1097-2765(00)80035-0

[17] Shen SX, Weaver Z, Xu X, Li C, Weinstein M, et al (1998) A targeted disruption of the murine Brca1 gene causes gamma-irradiation hypersensitivity and genetic instability. Oncogene 17: 3115–3124. 9872327 10.1038/sj.onc.1202243

[18] Alexandrov LB, Nik-Zainal S, Wedge DC, Aparicio SA, Behjati S, et al (1998) A targeted disruption of the murine Brca1 gene causes gamma-irradiation hypersensitivity and genetic instability. Oncogene 17: 3115–3124. 9872327 10.1038/sj.onc.1202243

[19] Kandoth C, McLellan MD, Vandin F, Ye K, Niu B, et al (2013) Mutational landscape and significance across 12 major cancer types. Nature 502: 333–339. 24132290 10.1038/nature12634 PMC3927368

[20] Agarwal R, Kaye SB (2003) Ovarian cancer: strategies for overcoming resistance to chemotherapy. Nat Rev Cancer 3: 502–516. 12835670 10.1038/nrc1123

[21] Taniguchi T, Tischkowitz M, Ameziane N, Hodgson SV, Mathew CG, et al (2003) Disruption of the Fanconi anemia-BRCA pathway in cisplatin-sensitive ovarian tumors. Nat Med 9: 568–574. 12692539 10.1038/nm852

[22] Cass I, Baldwin RL, Varkey T, Moslehi R, Narod SA, et al (2003) Improved survival in women with BRCA-associated ovarian carcinoma. Cancer 97: 2187–2195. 12712470 10.1002/cncr.11310

[23] Tan DS, Rothermundt C, Thomas K, Bancroft E, Eeles R, et al (2008) “BRCAness” syndrome in ovarian cancer: a case-control study describing the clinical features and outcome of patients with epithelial ovarian cancer associated with BRCA1 and BRCA2 mutations. J Clin Oncol 26: 5530–5536. 18955455 10.1200/JCO.2008.16.1703

[24] Lee JM, Ledermann JA, Kohn EC (2014) PARP Inhibitors for BRCA1/2 mutation-associated and BRCA-like malignancies. Ann Oncol 25: 32–40. 24225019 10.1093/annonc/mdt384 PMC3868320

[25] Farmer H, McCabe N, Lord CJ, Tutt AN, Johnson DA, et al (2005) Targeting the DNA repair defect in BRCA mutant cells as a therapeutic strategy. Nature 434: 917–921. 15829967 10.1038/nature03445

[26] Galluzzi L, Senovilla L, Vitale I, Michels J, Martins I, et al (2012) Molecular mechanisms of cisplatin resistance. Oncogene 31: 1869–1883. 21892204 10.1038/onc.2011.384

[27] Sakai W, Swisher EM, Karlan BY, Agarwal MK, Higgins J, et al (2008) Secondary mutations as a mechanism of cisplatin resistance in BRCA2-mutated cancers. Nature 451: 1116–1120. 18264087 10.1038/nature06633 PMC2577037

[28] Edwards SL, Brough R, Lord CJ, Natrajan R, Vatcheva R, et al (2008) Resistance to therapy caused by intragenic deletion in BRCA2. Nature 451: 1111–1115. 18264088 10.1038/nature06548

[29] Norquist B, Wurz KA, Pennil CC, Garcia R, Gross J, et al (2011) Secondary somatic mutations restoring BRCA1/2 predict chemotherapy resistance in hereditary ovarian carcinomas. J Clin Oncol 29: 3008–3015. 21709188 10.1200/JCO.2010.34.2980 PMC3157963

[30] Barber LJ, Sandhu S, Chen L, Campbell J, Kozarewa I, et al (2013) Secondary mutations in BRCA2 associated with clinical resistance to a PARP inhibitor. J Pathol 229: 422–429. 23165508 10.1002/path.4140

[31] Cooke SL, Ng CK, Melnyk N, Garcia MJ, Hardcastle T, et al (2010) Genomic analysis of genetic heterogeneity and evolution in high-grade serous ovarian carcinoma. Oncogene 29: 4905–4913. 20581869 10.1038/onc.2010.245 PMC2933510

[32] Iwasa Y, Nowak MA, Michor F (2006) Evolution of resistance during clonal expansion. Genetics 172: 2557–2566. 16636113 10.1534/genetics.105.049791 PMC1456382

[33] De Vargas Roditi L, Michor F (2011) Evolutionary dynamics of BRCA1 alterations in breast tumorigenesis. J Theor Biol 273: 207–215. 21194536 10.1016/j.jtbi.2010.12.033 PMC3713469

[34] Enderling H, Chaplain MA, Anderson AR, Vaidya JS (2007) A mathematical model of breast cancer development, local treatment and recurrence. J Theor Biol 246: 245–259. 17289081 10.1016/j.jtbi.2006.12.010

[35] Danesh K, Durrett R, Havrilesky LJ, Myers E (2012) A branching process model of ovarian cancer. J Theor Biol 314: 10–15. 22959913 10.1016/j.jtbi.2012.08.025 PMC3478401

[36] Foo J, Michor F (2010) Evolution of resistance to anti-cancer therapy during general dosing schedules. J Theor Biol 263: 179–188. 20004211 10.1016/j.jtbi.2009.11.022 PMC2826560

[37] Leder K, Foo J, Skaggs B, Gorre M, Sawyers CL, et al (2011) Fitness conferred by BCR-ABL kinase domain mutations determines the risk of pre-existing resistance in chronic myeloid leukemia. PLoS One 6: e27682. 22140458 10.1371/journal.pone.0027682 PMC3225363

[38] Diaz LA Jr, Williams RT, Wu J, Kinde I, Hecht JR, et al (2012) The molecular evolution of acquired resistance to targeted EGFR blockade in colorectal cancers. Nature 486: 537–540. 22722843 10.1038/nature11219 PMC3436069

[39] van Leeuwen IM, Byrne HM, Jensen OE, King JR (2006) Crypt dynamics and colorectal cancer: advances in mathematical modelling. Cell Prolif 39: 157–181. 16671995 10.1111/j.1365-2184.2006.00378.x PMC6495865

[40] Johnston MD, Edwards CM, Bodmer WF, Maini PK, Chapman SJ (2007) Mathematical modeling of cell population dynamics in the colonic crypt and in colorectal cancer. Proc Natl Acad Sci U S A 104: 4008–4013. 17360468 10.1073/pnas.0611179104 PMC1820699

[41] Komarova NL, Wodarz D (2007) Stochastic modeling of cellular colonies with quiescence: an application to drug resistance in cancer. Theor Popul Biol 72: 523–538. 17915274 10.1016/j.tpb.2007.08.003

[42] Beerenwinkel N, Antal T, Dingli D, Traulsen A, Kinzler KW, et al (2007) Genetic progression and the waiting time to cancer. PLoS Comput Biol 3: e225. 17997597 10.1371/journal.pcbi.0030225 PMC2065895

[43] Haeno H, Maruvka YE, Iwasa Y, Michor F (2013) Stochastic Tunneling of Two Mutations in a Population of Cancer Cells. PLoS One 8: e65724. 23840359 10.1371/journal.pone.0065724 PMC3694076

[44] Iijima J, Zeng Z, Takeda S, Taniguchi Y (2010) RAP80 acts independently of BRCA1 in repair of topoisomerase II poison-induced DNA damage. Cancer Res 70: 8467–8474. 20959489 10.1158/0008-5472.CAN-10-0267

[45] Lengauer C, Kinzler KW, Vogelstein B (1997) Genetic instability in colorectal cancers. Nature 386: 623–627. 9121588 10.1038/386623a0

[46] Kunkel TA, Bebenek K (2000) DNA replication fidelity. Annu Rev Biochem 69: 497–529. 10966467 10.1146/annurev.biochem.69.1.497

[47] Yamamoto KN, Kobayashi S, Tsuda M, Kurumizaka H, Takata M, et al (2011) Involvement of SLX4 in interstrand cross-link repair is regulated by the Fanconi anemia pathway. Proc Natl Acad Sci U S A 108: 6492–6496. 21464321 10.1073/pnas.1018487108 PMC3080998

[48] Khalique L, Ayhan A, Weale ME, Jacobs IJ, Ramus SJ, et al (2007) Genetic intra-tumour heterogeneity in epithelial ovarian cancer and its implications for molecular diagnosis of tumours. J Pathol 211: 286–295. 17154249 10.1002/path.2112

[49] Torres L, Ribeiro FR, Pandis N, Andersen JA, Heim S, et al (2007) Intratumor genomic heterogeneity in breast cancer with clonal divergence between primary carcinomas and lymph node metastases. Breast Cancer Res Treat 102: 143–155. 16906480 10.1007/s10549-006-9317-6

[50] Ahmed AA, Etemadmoghadam D, Temple J, Lynch AG, Riad M, et al (2010) Driver mutations in TP53 are ubiquitous in high grade serous carcinoma of the ovary. J Pathol 221: 49–56. 20229506 10.1002/path.2696 PMC3262968

[51] Liu G, Yang D, Sun Y, Shmulevich I, Xue F, et al (2012) Differing clinical impact of BRCA1 and BRCA2 mutations in serous ovarian cancer. Pharmacogenomics 13: 1523–1535. 23057551 10.2217/pgs.12.137 PMC3603383

[52] Noh JM, Han BK, Choi DH, Rhee SJ, Cho EY, et al (2013) Association between BRCA Mutation Status, Pathological Findings, and Magnetic Resonance Imaging Features in Patients with Breast Cancer at Risk for the Mutation. J Breast Cancer 16: 308–314. 24155760 10.4048/jbc.2013.16.3.308 PMC3800727

[53] Bayani J, Brenton JD, Macgregor PF, Beheshti B, Albert M, et al (2002) Parallel analysis of sporadic primary ovarian carcinomas by spectral karyotyping, comparative genomic hybridization, and expression microarrays. Cancer Res 62: 3466–3476. 12067990

[54] Jones R, Ryan M, Friedlander M (2003) Carboplatin hypersensitivity reactions: re-treatment with cisplatin desensitisation. Gynecologic Oncology 89: 112–115. 12694663 10.1016/s0090-8258(03)00066-0

[55] Ledermann J, Harter P, Gourley C, Friedlander M, Vergote I, et al (2012) Olaparib maintenance therapy in platinum-sensitive relapsed ovarian cancer. N Engl J Med 366: 1382–1392. 22452356 10.1056/NEJMoa1105535

[56] Omura GA, Brady MF, Homesley HD, Yordan E, Major FJ, et al (1991) Long-term follow-up and prognostic factor analysis in advanced ovarian carcinoma: the Gynecologic Oncology Group experience. J Clin Oncol 9: 1138–50. 1904477 10.1200/JCO.1991.9.7.1138

[57] Bayraktar S, Gluck S (2012) Systemic therapy options in BRCA mutation-associated breast cancer. Breast Cancer Res Treat 135: 355–366. 22791366 10.1007/s10549-012-2158-6

[58] Gelmon KA, Tischkowitz M, Mackay H, Swenerton K, Robidoux A, et al (2011) Olaparib in patients with recurrent high-grade serous or poorly differentiated ovarian carcinoma or triple-negative breast cancer: a phase 2, multicentre, open-label, non-randomised study. The Lancet Oncology 12: 852–861. 21862407 10.1016/S1470-2045(11)70214-5

[59] Shah SP, Roth A, Goya R, Oloumi A, Ha G, et al (2012) The clonal and mutational evolution spectrum of primary triple-negative breast cancers. Nature 486: 395–399. 22495314 10.1038/nature10933 PMC3863681

[60] Lord CJ, Ashworth A (2013) Mechanisms of resistance to therapies targeting BRCA-mutant cancers. Nat Med 19: 1381–1388. 24202391 10.1038/nm.3369

[61] Fong PC, Boss DS, Yap TA, Tutt A, Wu P, et al (2009) Inhibition of poly(ADP-ribose) polymerase in tumors from BRCA mutation carriers. N Engl J Med 361: 123–134. 19553641 10.1056/NEJMoa0900212

[62] Liu M, Mo QG, Wei CY, Qin QH, Huang Z, et al (2013) Platinum-based chemotherapy in triple-negative breast cancer: A meta-analysis. Oncol Lett 5: 983–991. 23426861 10.3892/ol.2012.1093 PMC3576281

[63] Kim A, Ueda Y, Naka T, Enomoto T (2012) Therapeutic strategies in epithelial ovarian cancer. J Exp Clin Cancer Res 31: 14. 22330607 10.1186/1756-9966-31-14 PMC3309949

[64] Dann RB, DeLoia JA, Timms KM, Zorn KK, Potter J, et al (2012) BRCA1/2 mutations and expression: response to platinum chemotherapy in patients with advanced stage epithelial ovarian cancer. Gynecol Oncol 125: 677–682. 22406760 10.1016/j.ygyno.2012.03.006

[65] Turner NC, Reis-Filho JS, Russell AM, Springall RJ, Ryder K, et al (2007) BRCA1 dysfunction in sporadic basal-like breast cancer. Oncogene 26: 2126–2132. 17016441 10.1038/sj.onc.1210014

[66] Wysham WZ, Mhawech-Fauceglia P, Li H, Hays L, Syriac S, et al (2012) BRCAness profile of sporadic ovarian cancer predicts disease recurrence. PLoS One 7: e30042. 22253870 10.1371/journal.pone.0030042 PMC3256213

[67] Saal LH, Gruvberger-Saal SK, Persson C, Lovgren K, Jumppanen M, et al (2008) Recurrent gross mutations of the PTEN tumor suppressor gene in breast cancers with deficient DSB repair. Nat Genet 40: 102–107. 18066063 10.1038/ng.2007.39 PMC3018354

