# Supplementary figures and images for: Evolution of Pre-Existing versus Acquired Resistance to Platinum Drugs and PARP Inhibitors in BRCA-Associated Cancers
Source: PLoS One. 2014 Aug 26;9(8):e105724. doi: 10.1371/journal.pone.0105724 (PMC4144917; doi:10.1371/journal.pone.0105724)

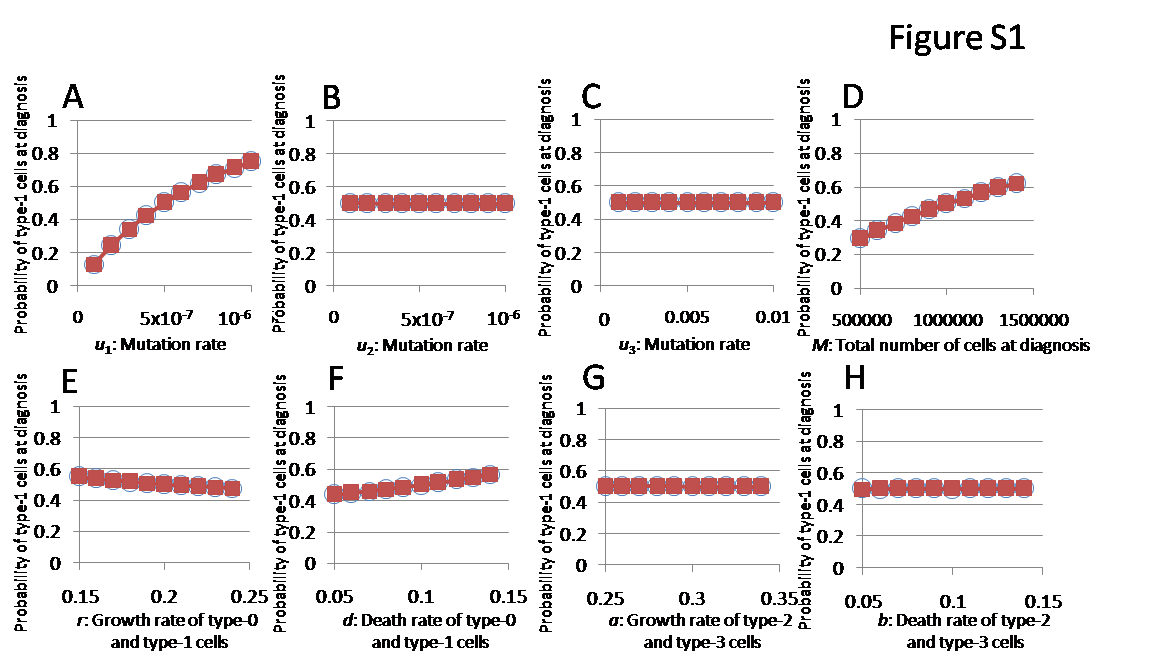

Supplement: Figure S1 — The probability of type-1 cells at diagnosis. The figure shows the dependence of the probability of the existence of type-1 cells at diagnosis on various parameters. The curves indicate the predictions of the analytical approximation, Eq. (1), while the circles indicate the results of the direct computer simulation (system S1). Standard parameter values used in the figure are u 1 = u 2 = 5.0⋅10−7, u 3 = 0.01, M = 106, r = 0.2, a = 0.3, d = b = 0.1. (TIF) [file pone.0105724.s001.tif]

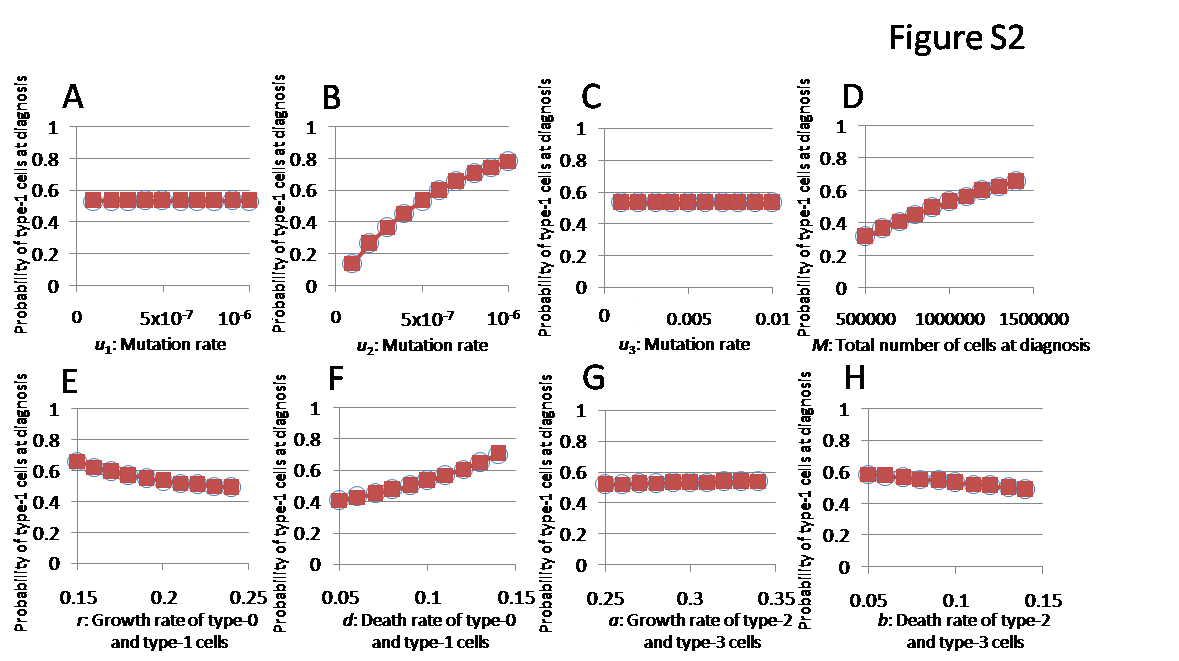

Supplement: Figure S2 — The probability of type-2 cells at diagnosis. The figure shows the dependence of the probability of the existence of type-2 cells at diagnosis on various parameters. The curves indicate the predictions of the analytical approximation, Eq. (2), while the circles indicate the results of the direct computer simulation (system S1). Standard parameter values used in the figure are u 1 = u 2 = 5.0⋅10−7, u 3 = 0.01, M = 106, r = 0.2, a = 0.3, d = b = 0.1. (TIF) [file pone.0105724.s002.tif]

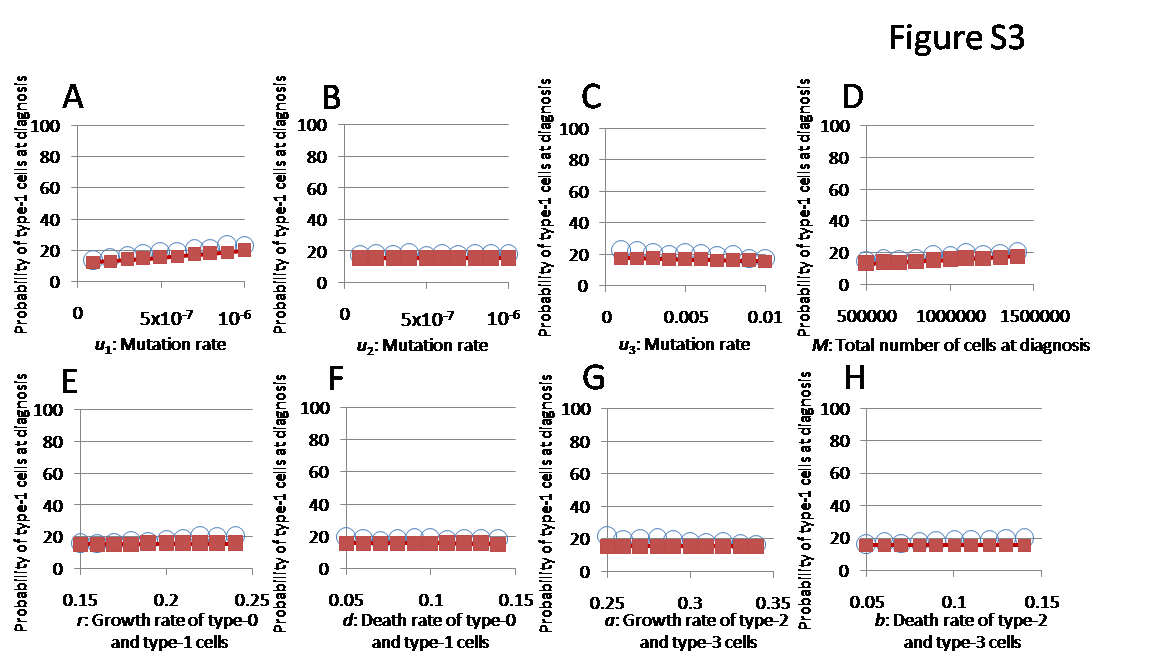

Supplement: Figure S3 — The expected number of type-1 cells at diagnosis. The figure shows the dependence of the expected number of type-1 cells at diagnosis on various parameters. The curves indicate the predictions of the analytical approximation, Eq. (S12), while the circles indicate the results of the direct computer simulation (system S1). Standard parameter values used in the figure are u 1 = u 2 = 5.0⋅10−7, u 3 = 0.01, M = 106, r = 0.2, a = 0.3, d = b = 0.1. (TIF) [file pone.0105724.s003.tif]

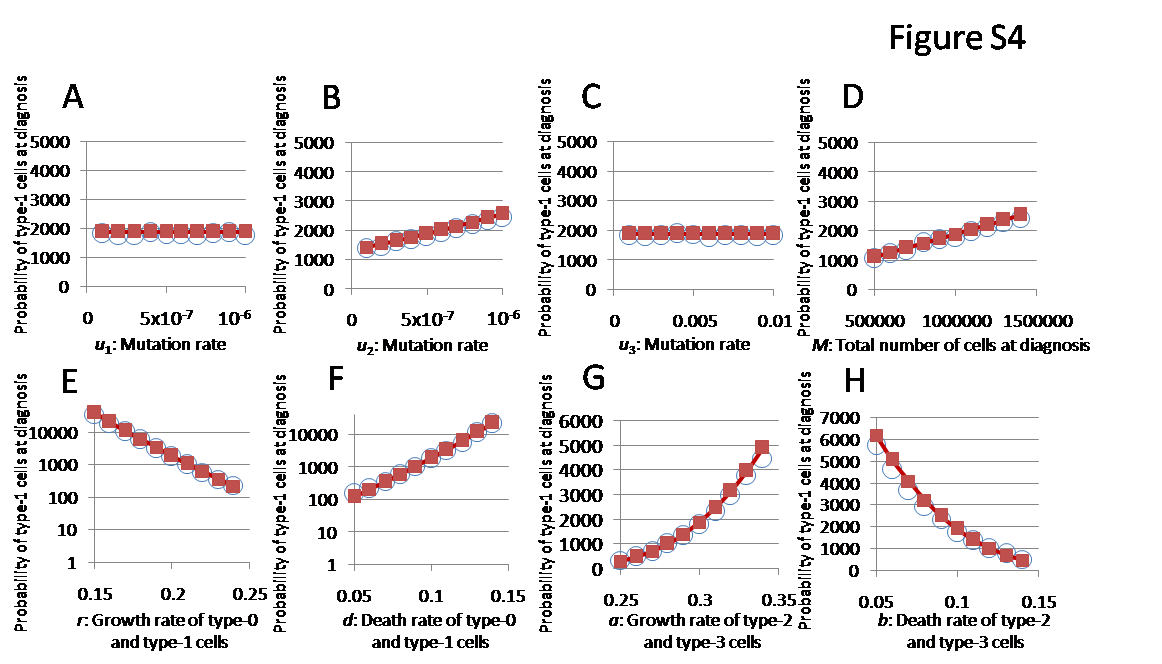

Supplement: Figure S4 — The expected number of type-2 cells at diagnosis. The figure shows the dependence of the expected number of type-2 cells at diagnosis on various parameters. The curves indicate the predictions of the analytical approximation, Eq. (S13), while the circles indicate the results of the direct computer simulation (system S1). Standard parameter values used in the figure are u 1 = u 2 = 5.0⋅10−7, u 3 = 0.01, M = 106, r = 0.2, a = 0.3, d = b = 0.1. (TIF) [file pone.0105724.s004.tif]

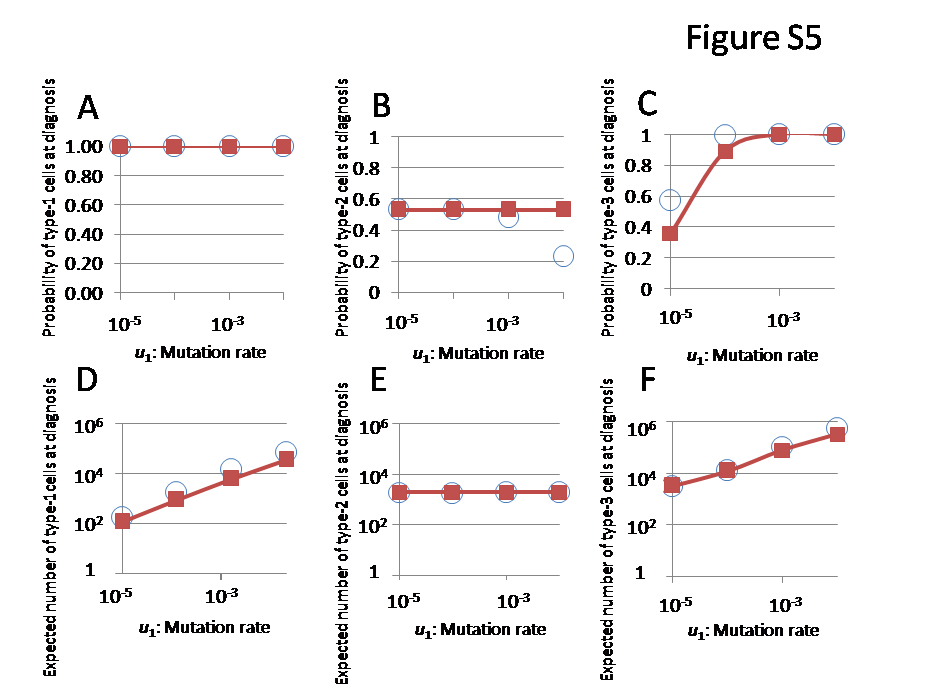

Supplement: Figure S5 — The probabilities and the expected numbers of each population at diagnosis with large u 1. The figure shows the probabilities of the existence of type-1, -2, and -3 cells and the expected numbers of type-1, -2, and -3 cells at diagnosis in a region of large u 1. The curves indicate the predictions of the analytical approximations, Eq. (1), Eq. (2), Eq. (S11), Eq. (S12), Eq. (S13), and Eq. (S22), while the circles indicate the results of the direct computer simulations (system S1). Parameter values used in the figure are u 2 = 5.0⋅10−7, u 3 = 0.01, M = 106, r = 0.2, a = 0.3, d = b = 0.1. (TIF) [file pone.0105724.s005.tif]

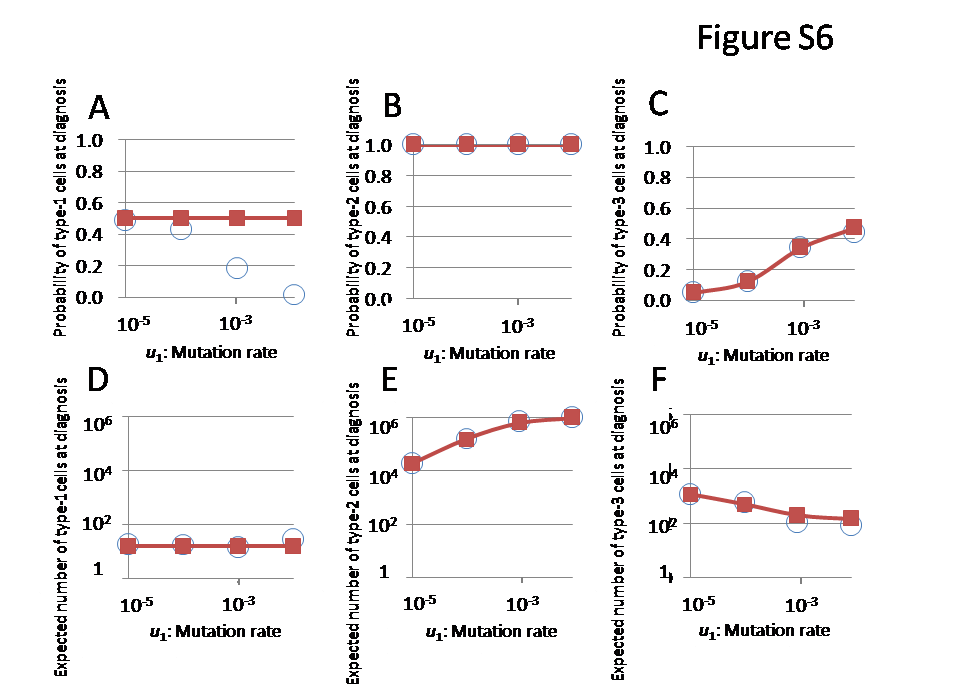

Supplement: Figure S6 — The probabilities and the expected numbers of each population at diagnosis with large u 2. The figure shows the probabilities of the existence of type-1, -2, and -3 cells and the expected numbers of type-1, -2, and -3 cells at diagnosis in a region of large u 2. The curves indicate the predictions of the analytical approximations, Eq. (1), Eq. (2), Eq. (S11), Eq. (S12), Eq. (S13), and Eq. (S22), while the circles indicate the results of the direct computer simulations (system S1). Parameter values used in the figure are u 1 = 5.0⋅10−7, u 3 = 0.01, M = 106, r = 0.2, a = 0.3, d = b = 0.1. (TIF) [file pone.0105724.s006.tif]

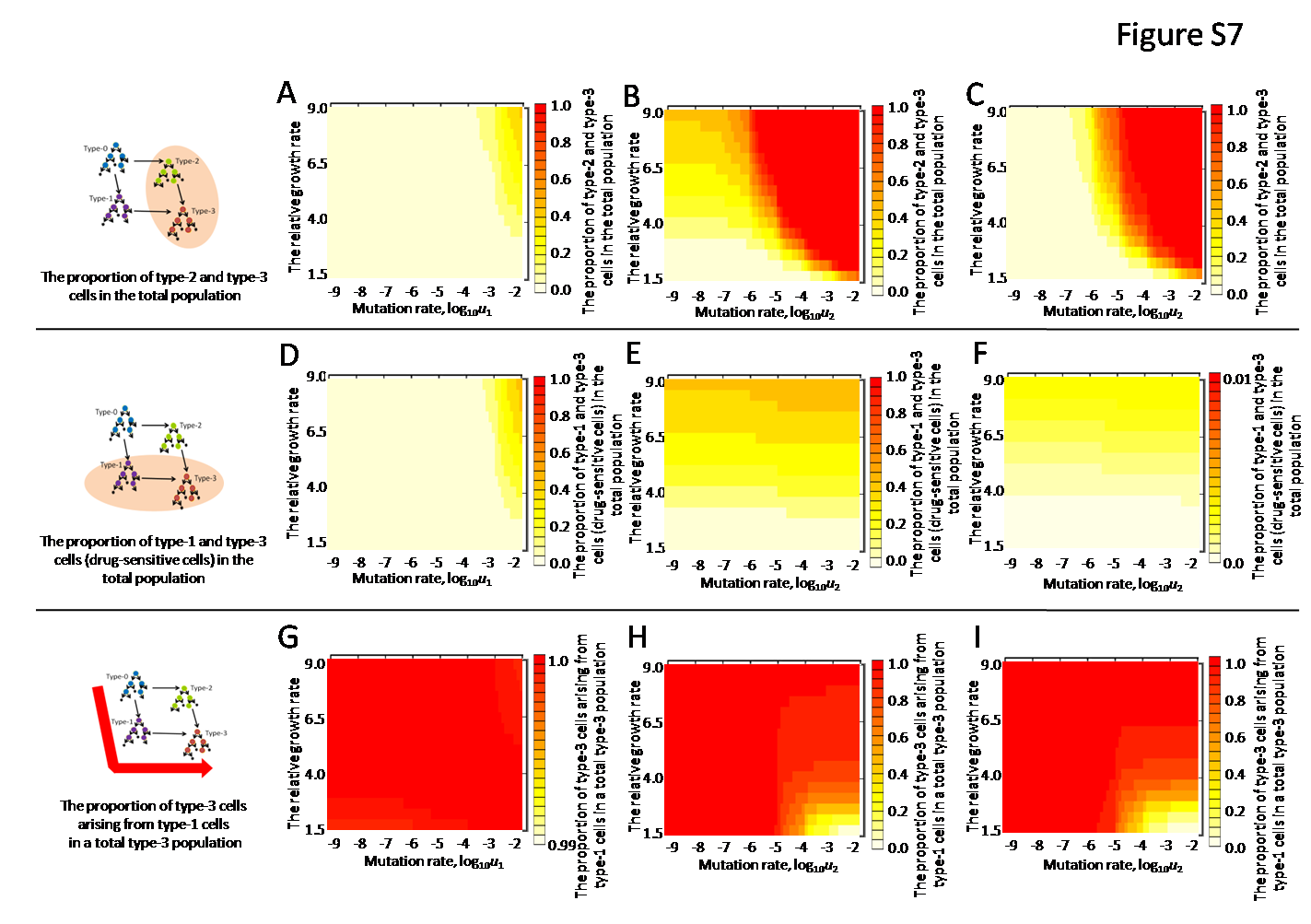

Supplement: Figure S7 — Proportion of clinically significant populations at diagnosis. (A–B) The proportion of type-2 and -3 cells with a growth advantage in the total population at diagnosis is shown in a wide region of u 1, u 2. (C-D) The proportion of type-1 and -3 cells (drug-sensitive cells) in the total population is shown. (E–F) The proportion of type-3 cells arising from type-1 cells in a total type-3 population is shown. Each population at diagnosis is calculated by the formulas, Eq. (S12), Eq. (S13), and Eq. (S22). Parameter values used in the figure are u 3 = 10−2, M = 106, r = 0.2, a = 0.3, d = b = 0.1 (panel A, C, and E); and a = 0.6 (panel B, D, and F). (TIF) [file pone.0105724.s007.tif]

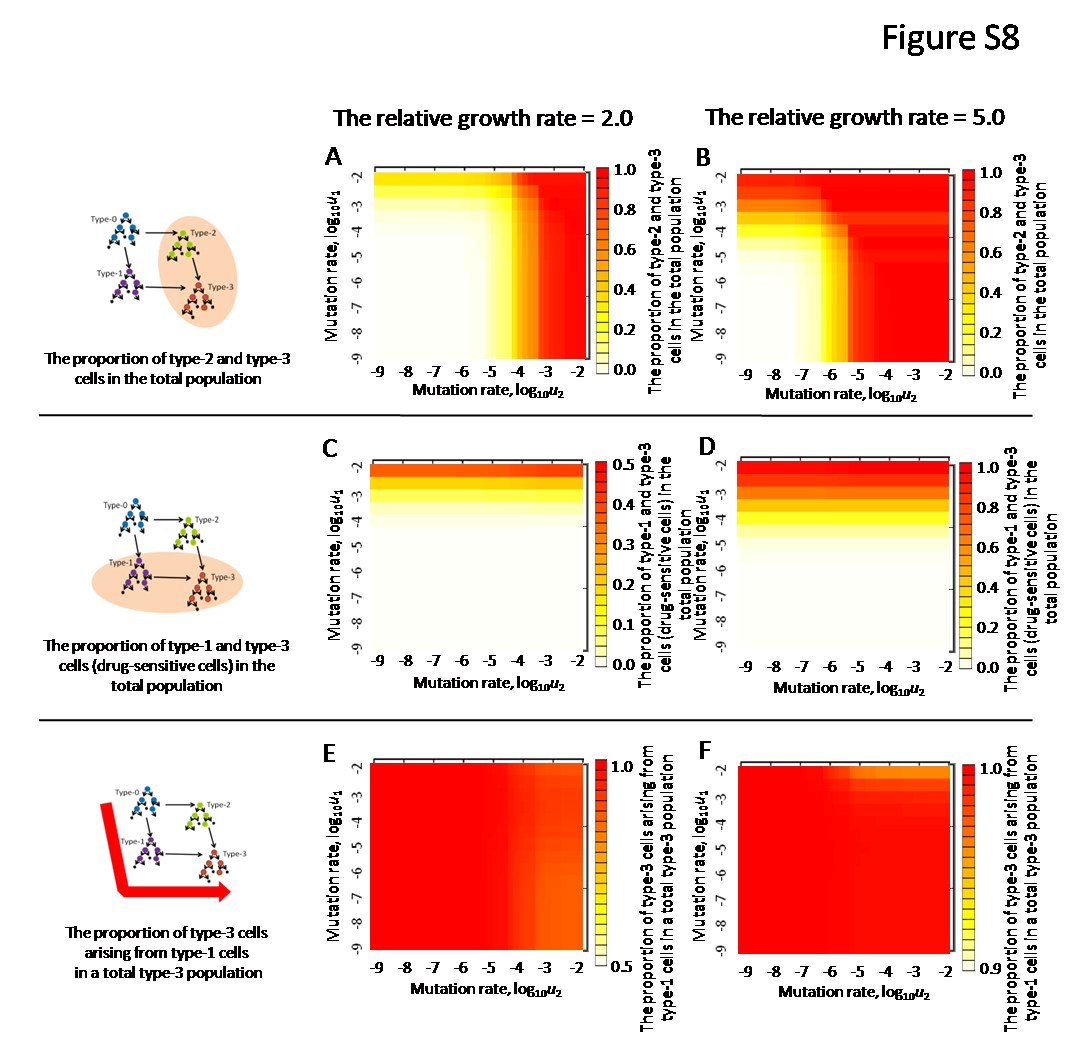

Supplement: Figure S8 — Proportion of clinically significant populations at diagnosis with a low mutation rate, u 3. (A–C) The proportion of type-2 and -3 cells with a growth advantage in the total population at diagnosis is shown in a wide region of u 1, u 2 and the relative growth rate of type-2 and -3 cells to that of type-0 and -1 cells, (a–b)/(r–d). (D–F) The proportion of type-1 and -3 cells (drug-sensitive cells) in the total population is shown. (G–I) The proportion of type-3 cells arising from type-1 cells in a total type-3 population is shown. Each population at diagnosis is calculated by the formulas, Eq. (S12), Eq. (S13), and Eq. (S22). Parameter values used in the figure are u 2 = 10−7, u 3 = 10−4, M = 106, r = 0.2, a = 0.3, d = b = 0.1 (panel A, D, and G); u 1 = 10−2 (panel B, E, and H); and u 1 = 10−7 (panel C, F, and I). (TIF) [file pone.0105724.s008.tif]

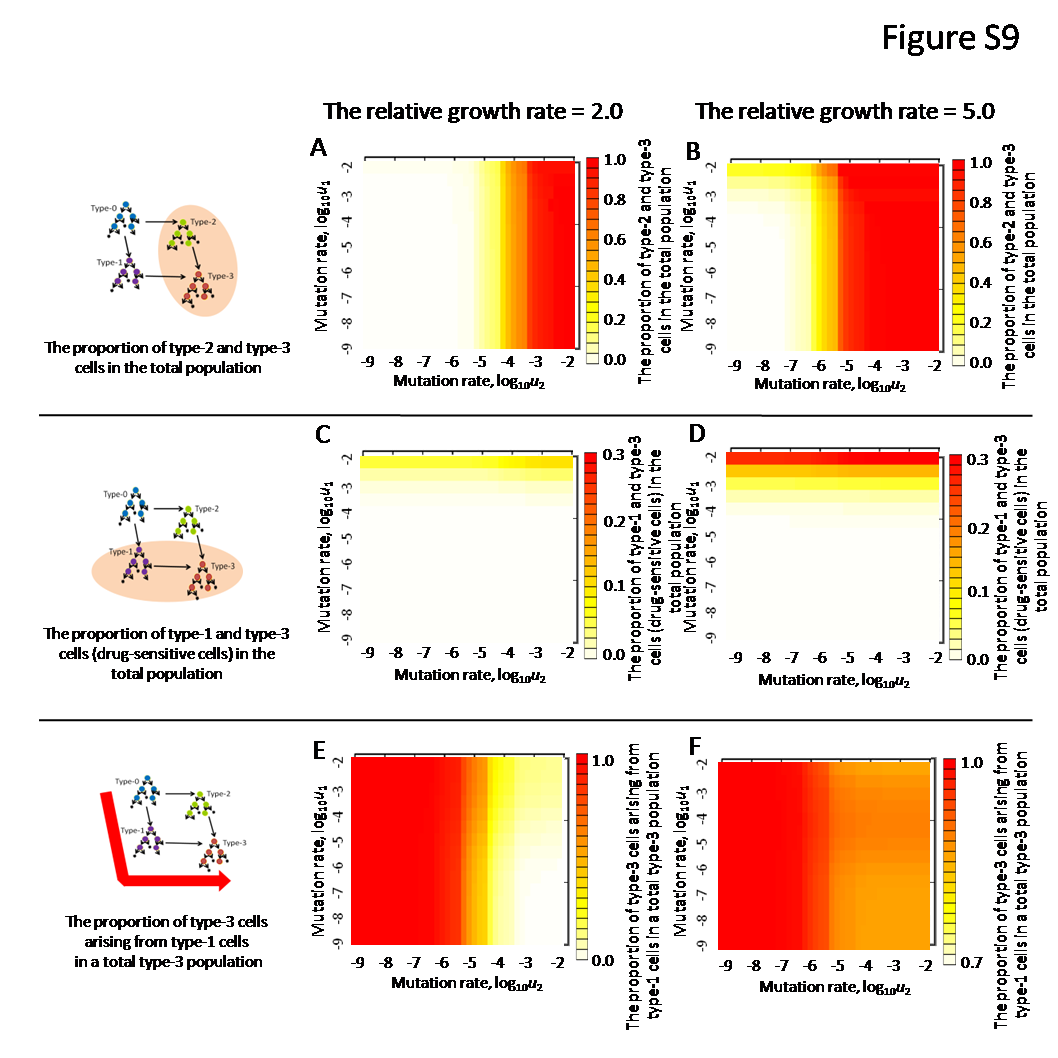

Supplement: Figure S9 — Proportion of clinically significant populations at diagnosis with a low mutation rate, u 3. (A–B) The proportion of type-2 and -3 cells with a growth advantage in the total population at diagnosis is shown in a wide region of u 1, u 2. (C–D) The proportion of type-1 and -3 cells (drug-sensitive cells) in the total population is shown. (E–F) The proportion of type-3 cells arising from type-1 cells in a total type-3 population is shown. Each population at diagnosis is calculated by the formulas, Eq. (S12), Eq. (S13), and Eq. (S22). Parameter values used in the figure are u 3 = 10−4, M = 106, r = 0.2, a = 0.3, d = b = 0.1 (panel A, C, and E); and a = 0.6 (panel B, D, and F). (TIF) [file pone.0105724.s009.tif]

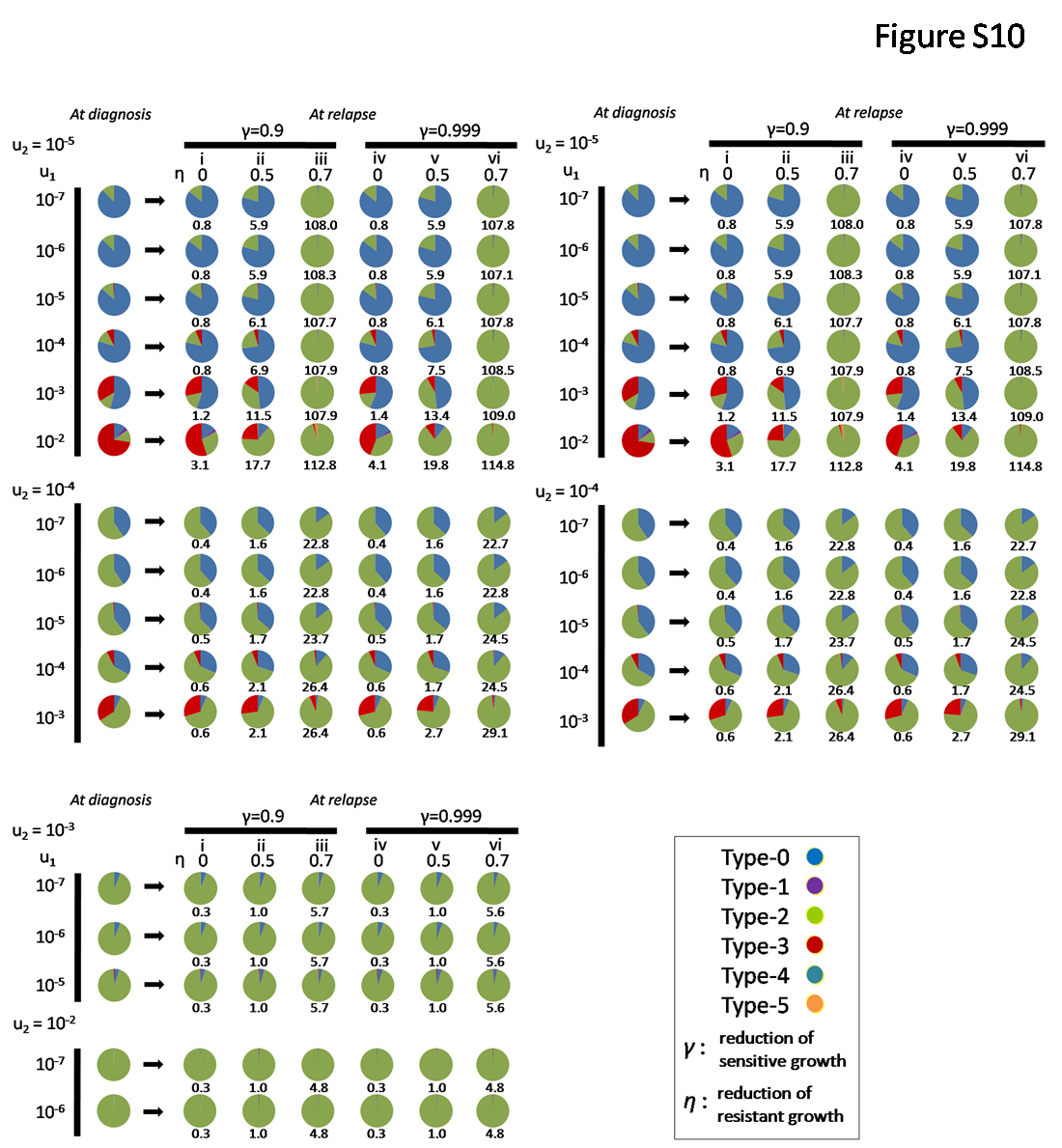

Supplement: Figure S10 — The population composition at relapse and recurrence time intervals in a wide region of u 1 and u 2. The population compositions at diagnosis (the initial time of treatment) and at the time of recurrence after treatment in a wide region of u 1 and u 2 are shown in pie charts. The time periods until the time of recurrence after treatment are shown as numbers under the pie charts. The time of recurrence is defined as the time point when the total number reaches 10% larger than the number at diagnosis. Each result is obtained by averaging a lot of trials by stochastic simulations of the model under treatment (system S23). The parameter values used in the figure except u 1 and u 2 are u 3 = u 4 = 0.01, M = 106, a = 0.4, and d = b = 0.1. Treatment effects are shown at the top of pie charts as the reduction effects on growth rates of sensitive populations (γ) and those on resistant populations (η). (TIF) [file pone.0105724.s010.tif]
