# Supplementary material for: Evolution of Pre-Existing versus Acquired Resistance to Platinum Drugs and PARP Inhibitors in BRCA-Associated Cancers
Source: PLoS One. 2014 Aug 26;9(8):e105724. doi: 10.1371/journal.pone.0105724 (PMC4144917; doi:10.1371/journal.pone.0105724)
Supplement: Materials S1 — Supplementary material. (DOCX) [file pone.0105724.s011.docx]

**SUPPLEMENTARY MATERIAL:**

**Evolution of pre-existing versus acquired resistance to platinum drugs and PARP inhibitors in BRCA-associated cancers**

Kimiyo N. Yamamoto, Kouji Hirota, Shunichi Takeda, and Hiroshi Haeno

**1. Computer simulations until diagnosis**

We perform exact computer simulations of the stochastic process. There are four types of cells: type-0, -1, -2 and -3 cells. Their respective numbers are denoted by *w*, *x*, *y*, and *z*. A change in the number of each type occurs by cell division (possibly with mutation) or by cell death. Initially, there is one type-0 cell, , and no mutant cell, . The stochastic simulation is performed by determining the probabilities of all possible events, the production or death of a type-0, -1, -2, or -3 cell. The chance of each event is proportional to its rate normalized by the sum of the rates of all possible events, given by $\Gamma=(r+d)(w+x)+(a+b)(y+z)$. The timing of the first event is given by an exponential distribution with mean $\frac{1}{\Gamma}$. The process is continued either until all cells go extinct, , or until the total cell number reaches the final size, , at diagnosis.

The transition probabilities between states of the stochastic process are determined as follows. The number of type-0 cells increases if a type-0 cell divides without mutation. Hence, the probability that the number of type-0 cells increases by one is given by

$\Pr[(w,x,y,z)\to(w+1,x,y,z)]=(rw(1-u_{1}-u_{2}))/\Gamma$. (S1a)

The number of type-1 cells increases by mutation of a type-0 cell or by division of a type-1 cell without mutation. The probability that the number of type-1 cells increases by one is given by

$\Pr[(w,x,y,z)\to(w,x+1,y,z)]=(rx(1-u_{3})+rwu_{1})/\Gamma$. (S1b)

The number of type-2 cells increases by mutation of a type-0 cell or by division of a type-2 cell without mutation. The probability that the number of type-2 cells increases by one is given by

$\Pr[(w,x,y,z)\to(w,x,y+1,z)]=(ay(1-u_{1})+rwu_{2})/\Gamma$. (S1c)

The number of type-3 cells increases by mutation of a type-1 or a type-2 cell, or by division of a type-3 cell. The probability that the number of type-3 cells increases by one is given by

$\Pr[(w,x,y,z)\to(w,x,y,z+1)]=(az+rxu_{3}+ayu_{1})/\Gamma$. (S1d)

The probabilities that the numbers of type-0, -1, -2, and -3 cells decrease by one are given by

$\Pr[(w,x,y,z)\to(w-1,x,y,z)]=wd/\Gamma$. (S1e)

$\Pr[(w,x,y,z)\to(w,x-1,y,z)]=xd/\Gamma$. (S1f)

$\Pr[(w,x,y,z)\to(w,x,y-1,z)]=yb/\Gamma$. (S1g)

$\Pr[(w,x,y,z)\to(w,x,y,z-1)]=zb/\Gamma$. (S1h)

For each parameter set, we perform many independent runs of the stochastic process to account for random fluctuations, and count the fraction of runs that reach the final size, *M*.

**2. The probability of the existence of type-3 cells at diagnosis**

Let us consider the probability that type-3 cells exist when total number of cells reaches *M*. Since we consider two types of mutations, there are two ways for the emergence of type-3 cells: (i) a mutation that increases a mutation rate occurs first and the other type of mutation occurs later; and (ii) a mutation that increases a net growth rate occurs first and the other mutation occurs later. Then, we first consider the former case. Two steps are separately calculated for the emergence of type-3 cells: (i) production of a type-1 cell by mutation and the survival of its lineage, and (ii) production of a type-3 cell from the lineage and its persistence. Denote by the probability that the first step occurs when there are *w* type-0 cells. Then we have

$P_{w}=e^{-u_{1}\frac{r}{d}\ln\frac{r}{r-d}(w-1)}\left(1-e^{-u_{1}\frac{r}{d}\ln\frac{r}{r-d}}\right)$. (S2)

Here $e^{-u_{1}\frac{r}{d}\ln\frac{r}{r-d}(w-1)}$ represents the probability that no successful type-1 lineage appears until total size becomes *w*. Then the population of cells contains *w* type-0 cells and one (eventually successful) type-1 cell. See Iwasa et al. (2006) and Haeno et al. (2007) [1,2] for the detailed derivation of $e^{-u_{1}\frac{r}{d}\ln\frac{r}{r-d}(w-1)}$. Denote by the probability of producing a type-3 cell from a successful type-1 lineage that arose when the number of type-0 cells is *w*. The total number of cell divisions the type-1 cell lineage experiences until the total population size is reached *M* is . Here is the abundance of cells emerging from such a successful type-1 lineage at diagnosis and $N_{1}=\exp[\left(r-d\right)\tau_{w}]$. is the time period from the emergence of a type-1 cell to the detection time and calculated by the following equation, ignoring the population of type-2 cells:

. (S3)

Then the probability of producing a type-3 cell from a type-1 cell lineage, , is given by

$Q_{w}=1-e^{-\frac{Mu_{3}F}{\left(w+1\right)\left(1-d/r\right)}}$. (S4)

Here, *F* represents the eventual survival probability of a type-3 lineage and given by

. (S5)

Please see Iwasa et al. 2006 for the detailed derivation of *F*. Finally the probability that there is at least one type-3 cell from a type-1 lineage when the total population size reaches *M* is given by

$P_{a}=\sum_{w=1}^{M-1} P_{w}\cdot Q_{w}=\sum_{w=1}^{M-1} e^{-u_{1}\frac{r}{d}\ln\frac{r}{r-d}(w-1)}\left(1-e^{-u_{1}\frac{r}{d}\ln\frac{r}{r-d}}\right)\left(1-\exp\left[-\frac{Mu_{3}F}{\left(w+1\right)\left(1-d/r\right)}\right]\right)$. (S6)

By considering the same process of derivation as Eq. (S6), the probability that there is at least one type-3 cell from a type-2 lineage when the total population size reaches *M* is given by

. (S7)

Here, the probability of the emergence of a successful type-2 cell when there are *w* type-0 cells, , is given by

$P'_{w}=e^{-\frac{u_{2}F\left(w-1\right)}{(1-d/r)}}\left(1-e^{-\frac{u_{2}F}{(1-d/r)}}\right)$. (S8)

Moreover, the probability of producing a type-3 cell from a successful type-2 lineage that arises when the number of type-0 cells is *w*, , is given by

$Q'_{w}=1-e^{-N_{2}u_{1}\frac{a}{b}\ln\frac{a}{a-b}}$. (S9)

Here, $N_{2}$ is the abundance of cells emerging from such a successful type-2 lineage at the time of diagnosis and $N_{2}=\exp[\left(a-b\right)\tau'_{w}]$. is numerically calculated by the following equation:

. (S10)

Finally the probability of the existence of type-3 cells at the detection time is given by

. (S11)

**3. The expected number of type-1, -2, and -3 cells at diagnosis**

Let us consider the expected number of type-1 cells when total number of cells reaches *M*. The expected number of type-1 cells conditional to type-1 cells having emerged at the detection time is given by

(S12)

Here, is the abundance of type-1 cells at the time of diagnosis. By considering exponential growth of type-1 cells, we have . Since the mutation rate from type-1 to -3 cells is considered to be relatively large, we excluded the cases of mutation from the growth of type-1 cells. Note that $P_{1}$, $P_{w}$, and $\tau_{w}$ have already been derived in Eq. (1), Eq. (S2) and Eq. (S3), respectively.

Let us next consider the expected number of type-2 cells when total number of cells reaches *M*, considering the same process of derivation as the expected number of type-1 cells. The expected number of type-2 cells conditional to type-2 cells having emerged at the time of diagnosis is given by

(S13)

Here $N_{2}=\exp[\left(a-b\right)\tau'_{w}]$ as shown in Appendix B. Note that , $P'_{w}$, and have already been derived in Eq. (2), Eq. (S8) and Eq. (S10), respectively.

Finally, let us consider the number of type-3 cells when the total number reaches *M*. Type-3 cells emerge through either type-1 or -2 cells. At first, let us consider the case in which type-1 cells produce type-3 cells. Note that we have already derived (i) the probability that a successful type-1 cell emerges when there are *w* type-0 cells, $P_{w}$, (ii) the period of time from type-1 arising from *w* type-0 cells to total number of type-0 and -1 cells reaching *M*, , and (iii) the probability that there is at least one type-3 cell at diagnosis, *P*_3_. Let us now consider the probability that a type-3 cell emerges at time $\sigma$ after the appearance of a type-1 cell. The probability that the first lineage of type-3 cells emerges at time $\sigma$ is given by

$L(\sigma)=\exp\left[-(e^{(r(1-u_{3})-d)\sigma}-1)\frac{ru_{3}}{r(1-u_{3})-d}\right]\left(1-\exp\left[-e^{(r(1-u_{3})-d)\sigma}ru_{3}\right]\right)$ (S14)

Here the probability that no type-3 cells have emerged until time $\sigma$ is given by $\exp\left[-\int_{0}^{\sigma} e^{(r(1-u_{3})-d)t}ru_{3}dt\right]$, leading to the first term in Eq. (S14), while the probability that a type-3 cell arises at time $\sigma$ is given by ($1-\exp\left[-e^{(r(1-u_{3})-d)\sigma}ru_{3}\right]$).

Once a type-3 cell has emerged, it gives rise to a lineage which increases exponentially until diagnosis. The time period between the emergence of the type-3 cell (which was produced from a lineage of type-1 cells that arose when there were w type-0 cells) and diagnosis is denoted by $\kappa_{w,\sigma}$, which is calculated from the following equation:

$we^{\left(r-d\right)(\sigma+\kappa_{w,\sigma})}+e^{\left(r-d\right)(\sigma+\kappa_{w,\sigma})}+e^{\left(a-b\right)\kappa_{w,\sigma}}=M$. (S15)

Then, the expected number of type-3 cells from a lineage of type-1 cells that arose when there were *w* type-0 cells at diagnosis, $S_{w}$ is given by

$S_{w}=\int_{0}^{\tau_{w}} L(\sigma)e^{(a-b)\kappa_{w,\sigma}}d\sigma$ (S16)

Hence, the expected number of type-3 cells produced from any lineages of type-1 cells at diagnosis is given by

(S17)

By considering the same process of derivation as Eq. (S17), the expected number of type-3 cells produced from any lineages of type-2 cells at diagnosis is given by

(S18)

Here the expected number of type-3 cells from a lineage of type-2 cells that arose when there were *w* type-0 cells at diagnosis is given by

(S19)

Here, the probability that the first lineage of type-3 cells from type-2 cells emerges at time $\sigma$, $L'(\sigma)$, and the amount of time between the emergence of the first type-3 cell (which was produced from a lineage of type-2 cells that arose when there were *w* type-0 cells) and diagnosis, , are given by

$L'(\sigma)=\exp\left[-(e^{(a-b)\sigma}-1)\frac{au_{1}}{a-b}\right]\left(1-\exp\left[-e^{(a-b)\sigma}au_{1}\right]\right)$ (S20)

(S21)

Note that we have already derived the probability that a successful type-2 cell emerges when there are *w* type-0 cells, $P'_{w}$, and the time of length in which a type-2 lineage created from *w* type-0 cells grows until total number of type-0 and -2 cells reaches *M*, $\tau'_{w}$. Finally, the expected number of type-3 cells at the detection time conditional to type-3 cells having emerged at diagnosis is given by

. (S22)

**4. Computer simulations under treatment**

We extend the model until diagnosis to incorporate two types of cells; type-4 and -5 cells which emerge at a mutation rate *u*_4_ by (epi)genetic alterations from either type-1 or -3 cells under treatment (Fig. 1B). Type-4 and -5 cells are resistant to platinum drugs and PARPis as type-0 and -2 cells are. Then, we investigate the population dynamics under treatment. The growth and death rates of type-4 cells are *r* and *d*, and those of type-5 cells are *a* and *b*. The respective numbers of type-4 and -5 are denoted by *i* and *j*. The reduction effects on growth rates of drug-sensitive and -resistant cells under treatment are given by *γ* and *η,* respectively. Note that each type of population exists at the start of treatment according to the parameter values and the expected numbers are calculated by the analytical equations derived above. We assume that neither type-4 nor -5 cells exist at the initial time of treatment.

The stochastic simulation is performed by determining the probabilities of all possible events, the production or death of a type-0, -1, -2, -3, -4, or -5 cell. The chance of each event is proportional to its rate normalized by the sum of the rates of all possible events, given by . The timing of the first event is given by an exponential distribution with mean . The process is continued until the detection of recurrence when the total cell number reaches 110% of the detection size, .

The transition probabilities between states of the stochastic process are determined as follows. The probability that the number of type-0 cells increases by one is given by

. (S23a)

The probability that the number of type-1 cells increases by one is given by

. (S23b)

The probability that the number of type-2 cells increases by one is given by

. (S23c)

The probability that the number of type-3 cells increases by one is given by

. (S23d)

The number of type-4 cells increases by a mutation of a type-1 cell or by a division of a type-4 cell. The probability that the number of type-4 cells increases by one is given by

. (S23e)

The number of type-5 cells increases by a mutation of a type-3 cell or by a division of a type-5 cell. The probability that the number of type-5 cells increases by one is given by

. (S23f)

The probabilities that the numbers of type-0, -1, -2, -3, -4 and -5 cells decrease by one are given by

. (S23g)

. (S23h)

. (S23i)

. (S23j)

. (S23k)

. (S23l)

For each parameter set, we perform many independent runs of the stochastic process to account for random fluctuations, and count the fraction of runs that reach the size of recurrence, . We also record the amount of time between diagnosis and recurrence.

**REFERENCES**

1. Iwasa Y, Nowak MA, Michor F (2006) Evolution of resistance during clonal expansion. Genetics 172: 2557-2566.

2. Haeno H, Iwasa Y, Michor F. (2007) The evolution of two mutations during clonal expansion. Genetics 177: 2209-2221.

**Figure S1. The probability of type-1 cells at diagnosis.**

The figure shows the dependence of the probability of the existence of type-1 cells at diagnosis on various parameters. The curves indicate the predictions of the analytical approximation, Eq. (1), while the circles indicate the results of the direct computer simulation (system S1). Standard parameter values used in the figure are *u*_1_ = *u*_2_ =$5.0\cdot10^{-7}$, *u*_3_ = 0.01, *M* = 10^6^, *r* = 0.2, *a* = 0.3, *d* = *b* = 0.1.

**Figure S2. The probability of type-2 cells at diagnosis.**

The figure shows the dependence of the probability of the existence of type-2 cells at diagnosis on various parameters. The curves indicate the predictions of the analytical approximation, Eq. (2), while the circles indicate the results of the direct computer simulation (system S1). Standard parameter values used in the figure are *u*_1_ = *u*_2_ =$5.0\cdot10^{-7}$, *u*_3_ = 0.01, *M* = 10^6^, *r* = 0.2, *a* = 0.3, *d* = *b* = 0.1.

**Figure S3. The expected number of type-1 cells at diagnosis.**

The figure shows the dependence of the expected number of type-1 cells at diagnosis on various parameters. The curves indicate the predictions of the analytical approximation, Eq. (S12), while the circles indicate the results of the direct computer simulation (system S1). Standard parameter values used in the figure are *u*_1_ = *u*_2_ =$5.0\cdot10^{-7}$, *u*_3_ = 0.01, *M* = 10^6^, *r* = 0.2, *a* = 0.3, *d* = *b* = 0.1.

**Figure S4. The expected number of type-2 cells at diagnosis.**

The figure shows the dependence of the expected number of type-2 cells at diagnosis on various parameters. The curves indicate the predictions of the analytical approximation, Eq. (S13), while the circles indicate the results of the direct computer simulation (system S1). Standard parameter values used in the figure are *u*_1_ = *u*_2_ =$5.0\cdot10^{-7}$, *u*_3_ = 0.01, *M* = 10^6^, *r* = 0.2, *a* = 0.3, *d* = *b* = 0.1.

**Figure S5. The probabilities and the expected numbers of each population at diagnosis with large *u*_1_.**

The figure shows the probabilities of the existence of type-1, -2, and -3 cells and the expected numbers of type-1, -2, and -3 cells at diagnosis in a region of large *u*_1_. The curves indicate the predictions of the analytical approximations, Eq. (1), Eq. (2), Eq. (S11), Eq. (S12), Eq. (S13), and Eq. (S22), while the circles indicate the results of the direct computer simulations (system S1). Parameter values used in the figure are *u*_2_ =$5.0\cdot10^{-7}$, *u*_3_ = 0.01, *M* = 10^6^, *r* = 0.2, *a* = 0.3, *d* = *b* = 0.1.

**Figure S6. The probabilities and the expected numbers of each population at diagnosis with large *u*_2_.**

The figure shows the probabilities of the existence of type-1, -2, and -3 cells and the expected numbers of type-1, -2, and -3 cells at diagnosis in a region of large *u*_2_. The curves indicate the predictions of the analytical approximations, Eq. (1), Eq. (2), Eq. (S11), Eq. (S12), Eq. (S13), and Eq. (S22), while the circles indicate the results of the direct computer simulations (system S1). Parameter values used in the figure are *u*_1_ =$5.0\cdot10^{-7}$, *u*_3_ = 0.01, *M* = 10^6^, *r* = 0.2, *a* = 0.3, *d* = *b* = 0.1.

**Figure S7. Proportion of clinically significant populations at diagnosis.**

(A-B) The proportion of type-2 and -3 cells with a growth advantage in the total population at diagnosis is shown in a wide region of *u*_1_, *u*_2_. (C-D) The proportion of type-1 and -3 cells (drug-sensitive cells) in the total population is shown. (E-F) The proportion of type-3 cells arising from type-1 cells in a total type-3 population is shown. Each population at diagnosis is calculated by the formulas, Eq. (S12), Eq. (S13), and Eq. (S22). Parameter values used in the figure are *u*_3_ = 10^-2^, *M* = 10^6^, *r* = 0.2, *a* = 0.3, *d* = *b* = 0.1 (panel A, C, and E); and *a* = 0.6 (panel B, D, and F).

**Figure S8. Proportion of clinically significant populations at diagnosis with a low mutation rate,** *u*_3_**.**

(A-C) The proportion of type-2 and -3 cells with a growth advantage in the total population at diagnosis is shown in a wide region of *u*_1_, *u*_2_ and the relative growth rate of type-2 and -3 cells to that of type-0 and -1 cells, . (D-F) The proportion of type-1 and -3 cells (drug-sensitive cells) in the total population is shown. (G-I) The proportion of type-3 cells arising from type-1 cells in a total type-3 population is shown. Each population at diagnosis is calculated by the formulas, Eq. (S12), Eq. (S13), and Eq. (S22). Parameter values used in the figure are *u*_2_ = 10^-7^, *u*_3_ = 10^-4^, *M* = 10^6^, *r* = 0.2, *a* = 0.3, *d* = *b* = 0.1 (panel A, D, and G); *u*_1_ = 10^-2^ (panel B, E, and H); and *u*_1_ = 10^-7^ (panel C, F, and I).

**Figure S9. Proportion of clinically significant populations at diagnosis with a low mutation rate,** *u*_3_**.**

(A-B) The proportion of type-2 and -3 cells with a growth advantage in the total population at diagnosis is shown in a wide region of *u*_1_, *u*_2_. (C-D) The proportion of type-1 and -3 cells (drug-sensitive cells) in the total population is shown. (E-F) The proportion of type-3 cells arising from type-1 cells in a total type-3 population is shown. Each population at diagnosis is calculated by the formulas, Eq. (S12), Eq. (S13), and Eq. (S22). Parameter values used in the figure are *u*_3_ = 10^-4^, *M* = 10^6^, *r* = 0.2, *a* = 0.3, *d* = *b* = 0.1 (panel A, C, and E); and *a* = 0.6 (panel B, D, and F).

**Figure S10. The population composition at relapse and recurrence time intervals in a wide region of *u*_1_ and *u*_2_.**

The population compositions at diagnosis (the initial time of treatment) and at the time of recurrence after treatment in a wide region of *u*_1_ and *u*_2_ are shown in pie charts. The time periods until the time of recurrence after treatment are shown as numbers under the pie charts. The time of recurrence is defined as the time point when the total number reaches 10% larger than the number at diagnosis. Each result is obtained by averaging a lot of trials by stochastic simulations of the model under treatment (system S23). The parameter values used in the figure except *u*_1_ and *u*_2_ are *u*_3_= *u*_4_ = 0.01, *M* = 10^6^, *a*=0.4, and *d* = *b* = 0.1. Treatment effects are shown at the top of pie charts as the reduction effects on growth rates of sensitive populations (*γ*) and those on resistant populations (*η*).

**Table S1. Parameter sets used for the analysis in Supplementary Figure S10 and expected numbers of cells at diagnosis.**

The expected numbers of type-1, -2, and -3 cells at diagnosis are calculated by Eq. (S12), Eq. (S13), and Eq. (S22). The number of type-0 cells is considered as the rest of the total number. The proportion of each type is shown in parentheses. The cases where the sum of type-0, type-1, type-2, and type-3 cells exceeded *M* were excluded. Parameter values used in the figure are *u*_3_ = 0.01, *M* = 10^6^, *r* = 0.2, *a* = 0.4, *d* = *b* = 0.1.
